# Joint associations of lipoprotein(a) and systemic inflammatory indices with FibroTouch-derived hepatic steatosis and liver stiffness in Chinese adults

**DOI:** 10.3389/fimmu.2026.1926199

**Published:** 2026-08-21

**Authors:** Dejin Huang, Zhonghua Sun, Xiaolong Shen, Tongtong Yu, Suying Liu, Ying Zhang, Wencong Li, Wei Zhang, Rongqi Wang

**Affiliations:** 1 Department of Traditional and Western Medical Hepatology, Hebei Provincial Key Laboratory of Liver Fibrosis in Chronic Liver Diseases, The Third Hospital of Hebei Medical University, Shijiazhuang, China; 2 Medical School, Nanjing University, Nanjing, China; 3 Health Management Center, Baoding People’s Hospital, Baoding, China

**Keywords:** lipoprotein(a), liver stiffness, nonalcoholic fatty liver disease, SII, SIRI, systemic inflammation

## Abstract

**Background:**

Lipoprotein(a) [Lp(a)] is synthesized mainly in the liver, whereas blood cell-derived inflammatory indices reflect systemic immune–inflammatory status. We examined their associations with FibroTouch-assessed hepatic steatosis and liver stiffness in Chinese adults.

**Methods:**

This hospital-based cross-sectional study included 1,345 Chinese adults. Nonalcoholic fatty liver disease (NAFLD) was defined as controlled attenuation parameter (CAP) ≥248 dB/m, and elevated liver stiffness as liver stiffness measurement (LSM) ≥8 kPa. Multivariable logistic and linear regression, restricted cubic spline (RCS), receiver operating characteristic (ROC), joint-exposure, subgroup, and sensitivity analyses were performed.

**Results:**

Higher Lp(a) quartiles were associated with lower prevalences of NAFLD and elevated liver stiffness and with lower CAP and LSM values. After full adjustment, the highest versus lowest quartile was associated with lower odds of NAFLD (odds ratio [OR], 0.28; 95% confidence interval [CI], 0.20–0.41) and elevated liver stiffness (OR, 0.31; 95% CI, 0.21–0.45). RCS analyses showed significant inverse associations without evidence of nonlinearity. Higher Lp(a) was also associated with lower CAP (β, −14.40; 95% CI, −17.79 to −11.01) and LSM (β, −0.58; 95% CI, −0.83 to −0.33). The Lp(a)–systemic immune-inflammation index (SII) combination showed the highest discrimination for NAFLD, whereas Lp(a)–systemic inflammation response index (SIRI) performed best for elevated liver stiffness. When added to the fully adjusted model, the corresponding areas under the ROC curve (AUCs) were 0.762 and 0.811, respectively, and exceeded those of established noninvasive fibrosis scores. Low Lp(a) combined with high inflammatory burden was associated with the highest odds of both outcomes.

**Conclusions:**

Among Chinese adults, higher circulating Lp(a) was inversely associated with FibroTouch-derived hepatic steatosis and liver stiffness. Combining Lp(a) with systemic inflammatory indices may provide complementary information for noninvasive cross-sectional assessment.

## 1 Introduction

Nonalcoholic fatty liver disease (NAFLD) represents a highly prevalent chronic liver condition and has increasingly become a key concern in the management of metabolic health. Its pathological spectrum extends from simple lipid deposition in the liver to inflammatory injury, fibrotic remodeling, cirrhosis, and hepatocellular carcinoma (1). NAFLD commonly coexists with obesity, type 2 diabetes, abnormal lipid profiles, hypertension, and cardiovascular disease, indicating its close relationship with systemic metabolic dysfunction (2). Hepatic fibrosis is a key histological feature of NAFLD and plays a central role in determining long-term liver prognosis. Therefore, timely recognition of liver fat accumulation and fibrotic change is essential. Although biopsy provides direct pathological information, its invasiveness limits widespread use in routine screening. Noninvasive imaging techniques are useful alternatives, but their availability may vary across clinical settings. For this reason, easily accessible circulating biomarkers are increasingly being explored as supplementary tools for the noninvasive assessment of prevalent liver disease.

Lipoprotein(a) [Lp(a)] is primarily synthesized in the liver and consists of an LDL-like particle containing apolipoprotein B-100 that is covalently linked to apolipoprotein(a). While Lp(a) is widely recognized as a largely genetically regulated contributor to cardiovascular risk, its hepatic synthesis has also attracted interest in liver-related research. Serum Lp(a) may reflect hepatic lipid regulation, hepatocellular functional status, and protein synthetic capacity (3, 4). Previous studies have reported lower Lp(a) concentrations among individuals with fatty liver, steatohepatitis, or fibrotic liver injury (5, 6). Population-level evidence has also supported an inverse linear association between Lp(a) concentration and the presence of NAFLD (7). These findings imply that Lp(a) may have relevance not only in cardiovascular evaluation but also in liver disease assessment. Nevertheless, evidence from Chinese clinical populations remains limited, especially from studies using quantitative noninvasive methods to assess hepatic fat burden and stiffness simultaneously.

Inflammation is closely involved in the pathophysiological processes associated with NAFLD and greater disease severity. In routine practice, systemic immune–inflammatory activity can be indirectly assessed through standard blood test results. Blood cell-derived indices, including the systemic inflammation response index (SIRI), systemic immune-inflammation index (SII), and aggregate index of systemic inflammation (AISI), summarize inflammatory information from platelets, neutrophils, monocytes, and lymphocytes. Compared with single blood cell parameters, these indices may provide a broader picture of systemic inflammatory imbalance (8–12). Earlier studies have reported associations of these markers with fatty liver disease, fibrosis-related measures, and unfavorable clinical outcomes (13, 14). Because they are inexpensive, easy to calculate, and widely available, these indices may help distinguish individuals who may warrant further liver imaging or stiffness assessment.

However, NAFLD and elevated liver stiffness are complex phenotypes that may not be adequately characterized by a single lipid-related factor or inflammatory marker. Lp(a) may provide information on liver-derived lipoprotein metabolism and hepatic synthetic activity, whereas blood cell-based indices may reflect systemic immune activation. Assessing these markers together could therefore provide complementary information for discriminating prevalent NAFLD and distinguishing individuals with higher liver stiffness values. At present, few studies have jointly examined Lp(a), SIRI, SII, and AISI in Chinese adults. Additionally, the associations of Lp(a) with FibroTouch-derived controlled attenuation parameter (CAP) and liver stiffness measurement (LSM) values analyzed as continuous variables remain insufficiently investigated.

A total of 1,345 Chinese adults were evaluated in this study. FibroTouch examination provided CAP and LSM measurements, which were used to quantify hepatic fat deposition and stiffness. These outcomes were analyzed according to predefined clinical thresholds and as continuous variables. We examined the associations of Lp(a) and systemic inflammatory indices with prevalent NAFLD, NAFLD with elevated liver stiffness, CAP, and LSM. We further assessed whether models combining Lp(a) with inflammatory indices showed greater discriminatory ability for prevalent NAFLD and elevated liver stiffness than individual markers and established noninvasive fibrosis scores.

## 2 Materials and methods

### 2.1 Study population

This hospital-based cross-sectional study enrolled adults attending the Department of Traditional and Western Medical Hepatology or undergoing routine health examinations at the Third Hospital of Hebei Medical University between October 2025 and April 2026. All participants underwent biochemical testing and FibroTouch examination.

**Figure 1:**
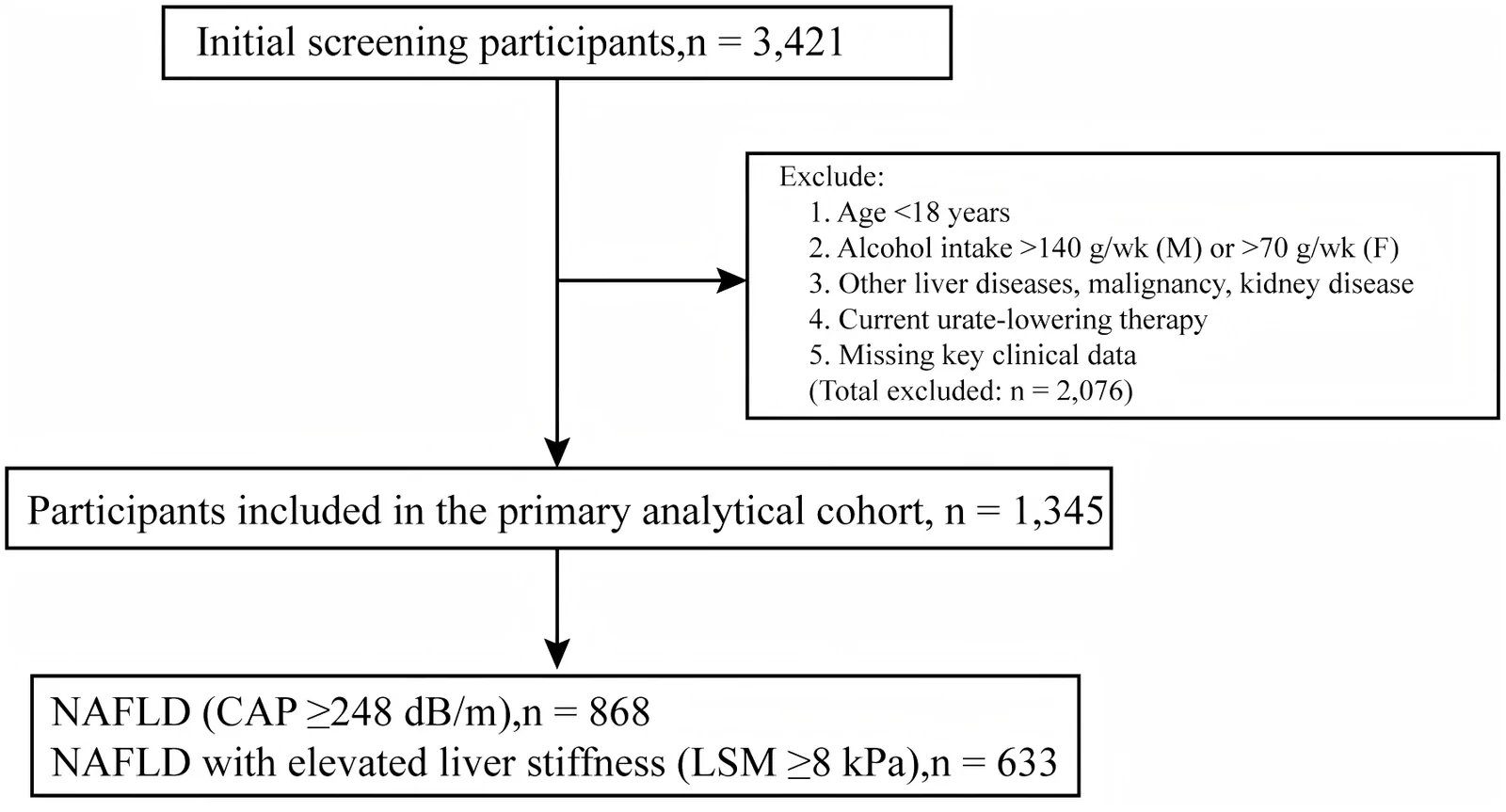
Flowchart of participant selection.

Of 3,421 individuals screened, participants were excluded for age <18 years, excessive alcohol consumption, secondary hepatic steatosis, malignancy or kidney disease, urate-lowering therapy, or missing essential data. The primary complete-case analysis included 1,345 participants. An additional multiple-imputation analysis included 2,349 otherwise eligible participants with valid Lp(a), inflammatory-index, CAP, and LSM measurements (**Figure 1**).

Written informed consent was obtained from all participants before enrollment. The study protocol was approved by the Human Research Ethics Committee of the Third Hospital of Hebei Medical University (Approval No. 2024-141-1). All study procedures were conducted in accordance with the ethical principles of the Declaration of Helsinki.

### 2.2 Diagnostic criteria and definitions

Hepatic steatosis and liver stiffness were assessed using FibroTouch in accordance with the 2024 Chinese guideline for metabolic dysfunction-associated fatty liver disease (15). Hepatic fat accumulation and liver stiffness were quantified using the controlled attenuation parameter (CAP) and liver stiffness measurement (LSM), respectively. In the primary analysis, NAFLD was defined as CAP ≥248 dB/m, a biopsy-referenced threshold identified by Karlas et al. for detecting histological steatosis grade ≥S1 (16). An LSM threshold of ≥8 kPa was used to identify participants with elevated liver stiffness or a positive stiffness-based risk signal (17), and NAFLD with elevated liver stiffness was defined as the simultaneous presence of CAP ≥248 dB/m and LSM ≥8 kPa.

Body mass index (BMI) was calculated as weight in kilograms divided by height in meters squared (18). Dyslipidemia was defined according to the criteria recommended in the 2024 Chinese primary-care guideline for lipid management. Participants were classified as having dyslipidemia if they met at least one of the following criteria: total cholesterol (TC) ≥6.2 mmol/L, triglycerides (TG) ≥2.3 mmol/L, low-density lipoprotein cholesterol (LDL-C) ≥4.1 mmol/L, or high-density lipoprotein cholesterol (HDL-C) <1.0 mmol/L (19).

Diabetes mellitus was defined as fasting plasma glucose (FPG) ≥7.0 mmol/L, 2-hour plasma glucose ≥11.1 mmol/L during an oral glucose tolerance test, or a previous physician diagnosis of diabetes (20).

Hypertension was defined as a mean SBP ≥140 mmHg and/or mean DBP ≥90 mmHg, a previous physician diagnosis of hypertension, or current antihypertensive medication use (21).

### 2.3 Clinical data collection and measurements

#### 2.3.1 Collection of general information

Trained physicians administered a structured questionnaire to collect demographic characteristics, lifestyle factors, medication use, and medical history (**Supplementary Figure 1**).

#### 2.3.2 Physical examination

Anthropometric measurements and blood pressure assessments were performed by trained nurses according to a standardized protocol. Standing height was measured without shoes using a stadiometer, with participants maintaining an upright posture and their heads positioned in the Frankfort horizontal plane. Body weight was measured while participants wore light clothing and no shoes. Height and weight were recorded to the nearest 0.1 cm and 0.1 kg, respectively. Each measurement was obtained twice, and the mean value was used in the analyses.

Blood pressure was measured using an electronic upper-arm sphygmomanometer. Participants were instructed to remain seated quietly for at least 5 minutes before measurement, with their backs supported and their arms positioned at heart level. Three consecutive measurements were obtained at 1-minute intervals, and the mean of the three readings was used in the analyses.

#### 2.3.3 Laboratory tests

After an overnight fast, peripheral venous blood samples were collected from each participant in the morning. Serum biochemical measurements and routine hematological testing were performed separately in the hospital clinical laboratory under standardized quality-control procedures.

The serum biochemical variables included TG, TC, HDL-C, LDL-C, FPG, alanine aminotransferase (ALT), aspartate aminotransferase (AST), gamma-glutamyl transferase (GGT), total protein (TP), albumin (ALB), globulin (GLB), total bilirubin (TBIL), direct bilirubin (DBIL), indirect bilirubin (IBIL), uric acid (UA), and high-sensitivity C-reactive protein (hs-CRP). These biochemical variables were measured using standard laboratory methods with a Beckman Coulter AU5800 automated biochemical analyzer.

Routine hematological testing provided white blood cell (WBC), neutrophil (NEU), lymphocyte (LYM), monocyte (MONO), and platelet (PLT) counts.

Renal function was assessed using the estimated glomerular filtration rate, which was calculated from serum creatinine, age, and sex according to the 2021 Chronic Kidney Disease Epidemiology Collaboration creatinine equation without a race coefficient:


$$\text{eGFR}=142\times \min{\left(\frac{\text{Scr}}{\kappa },1\right)}^{\alpha }\times \max{\left(\frac{\text{Scr}}{\kappa },1\right)}^{-1.200}\times 0.9938^{\text{Age}}\times 1.012\;(iffemale)$$

κ is 0.7 for women and 0.9 for men, and α is −0.241 for women and −0.302 for men. The calculated estimated glomerular filtration rate (eGFR) was expressed in mL/min/1.73 m² (22).

Fibrosis-4 index (FIB-4), aspartate aminotransferase-to-platelet ratio index (APRI), and NAFLD fibrosis score (NFS) were calculated using the following formulas:


$$\text{FIB}-4=\frac{\text{Age}\times \text{AST}}{\text{PLT}\times \sqrt{\text{ALT}}}$$

$$\text{APRI}=\frac{\text{AST}/{\text{ULN}}_{\text{AST}}}{\text{PLT}}\times 100$$

$$\text{NFS}=-1.675+0.037\times \text{Age}+0.094\times \text{BMI}+1.13\times \text{IFG}/\text{diabetes}+0.99\times \frac{\text{AST}}{\text{ALT}}-0.013\times \text{PLT}-0.66\times \text{Albumin}$$

IFG/diabetes was coded as 1 when present and 0 when absent. PLT was expressed in 10^9^/L, and albumin was expressed in g/dL.

#### 2.3.4 Assessment of exposure variables

Serum Lp(a) was measured using an immunoturbidimetric assay and expressed in mg/L. SIRI, SII, and AISI were calculated from absolute blood-cell counts as follows (23–25):


$$\text{SIRI}=\frac{neutrophilcount\times monocytecount}{lymphocytecount}$$

$$\text{SII}=\frac{plateletcount\times neutrophilcount}{lymphocytecount}$$

$$\text{AISI}=\frac{neutrophilcount\times plateletcount\times monocytecount}{lymphocytecount}$$

#### 2.3.5 Imaging assessment

Liver transient elastography was performed using a FibroTouch system after an overnight fast. Participants were examined in the supine position. CAP and LSM values were obtained during the same examination.

Repeated measurements were performed until 10 valid readings had been obtained for each participant, and the median value was used in the statistical analyses. Measurements were considered reliable only when the success rate was >90% and the interquartile range-to-median ratio was <0.20. All FibroTouch examinations were performed by the same trained physician to maintain procedural consistency.

### 2.4 Statistical analysis

Statistical analyses were performed using R version 4.5.2 and IBM SPSS Statistics version 22.0. Graphs were generated with GraphPad Prism version 10.1.2 and edited using Adobe Illustrator version 29.7.1. Normally distributed variables were expressed as mean ± standard deviation and compared using one-way analysis of variance. Non-normally distributed variables were expressed as median (interquartile range) and compared using the Kruskal–Wallis test. Categorical variables were presented as n (%) and compared using the chi-square or Fisher’s exact test, as appropriate.

Before multivariable logistic regression, exploratory mediation and collinearity assessments were performed for the laboratory variables in the fully adjusted model. Selected biochemical markers were examined as potential mediators of the cross-sectional association between Lp(a) and prevalent NAFLD after adjustment for age, sex, hypertension, and BMI. Indirect effects were estimated using percentile bootstrap confidence intervals (CIs). Pearson correlation coefficients were used to assess pairwise correlations between laboratory variables and the inflammatory indices, while variance inflation factors (VIFs) and tolerance values were used to evaluate multicollinearity. Prespecified conventional criteria were applied to determine substantial correlation and acceptable collinearity.

Logistic regression was used to evaluate the associations of Lp(a) with NAFLD and NAFLD with elevated liver stiffness. Lp(a) was analyzed as quartiles and as a standardized continuous variable; for continuous analyses, odds ratios (ORs) represented the change in the odds of the prevalent outcome per 1-standard-deviation increase in Lp(a). The lowest Lp(a) quartile was used as the reference. Three models were constructed: Model 1 was unadjusted; Model 2 was adjusted for age, sex, hypertension, and BMI; and Model 3 was further adjusted for diabetes, ALT, AST, TC, TG, HDL-C, LDL-C, UA, eGFR, GGT, ALB, GLB, DBIL, and IBIL.

Restricted cubic spline (RCS) models with knots at the 5th, 35th, 65th, and 95th percentiles examined potential nonlinear associations, using the median Lp(a) concentration as the reference and Model 3 covariate adjustment (26, 27).

Receiver operating characteristic (ROC) curve analyses assessed the discriminatory ability of Lp(a), SIRI, SII, AISI, and biomarker-only combinations for prevalent NAFLD and NAFLD with elevated liver stiffness. Because lower Lp(a) levels were associated with higher outcome odds, Lp(a) was analyzed in the reverse direction. Combined-model probabilities were derived from logistic regression models including Lp(a) and one inflammatory index. Optimal cutoffs were identified using the Youden index, with accuracy, sensitivity, specificity, positive predictive value, and negative predictive value calculated.

For multivariable evaluation, each biomarker and biomarker combination was added separately to fully adjusted Model 3, with the FIB-4, APRI, and NFS included as comparators. The area under the ROC curve (AUC), standard error, Youden index, sensitivity, specificity, and optimal cutoff were calculated. Incremental value beyond Model 3 was assessed using integrated discrimination improvement (IDI) and net reclassification improvement (NRI), with 95% confidence intervals.

For the joint-exposure analyses, Lp(a) and each inflammatory index were dichotomized according to their respective median values. Values below the median were classified as low, whereas values equal to or above the median were classified as high. The median cutoffs were 277 mg/L for Lp(a), 0.83 for SIRI, 450.33 for SII, and 162.86 for AISI. Because higher Lp(a) was associated with lower odds of the prevalent liver-related outcomes, whereas higher inflammatory index levels were associated with higher odds, the high-Lp(a)/low-inflammation category was selected as the reference group to facilitate interpretation of the joint associations. Multiplicative interaction was assessed by including cross-product terms in the regression models.

Stratified analyses were performed across predefined demographic and clinical subgroups to examine the consistency of the observed cross-sectional associations. In the subgroup analyses, ORs were estimated per 1-standard-deviation increase in Lp(a) using Model 3, with the stratifying variable excluded from the corresponding model.

Model-specific complete-case analysis was used for the primary regression analyses. As a sensitivity analysis, missing covariate data were handled using multiple imputation by chained equations. Lp(a), SIRI, SII, AISI, CAP, LSM, and the derived outcome variables were not imputed. Age and sex were fully observed and therefore were also not imputed. All variables included in the corresponding analytical models, including the non-imputed exposure and outcome variables, were incorporated into the imputation models. Thirty imputed datasets were generated. The logistic regression analyses defining elevated Lp(a) as ≥300 mg/L were repeated in each imputed dataset, and the resulting estimates were pooled using Rubin’s rules.

Continuous variables were screened for implausible values using range and clinical plausibility checks, while biologically plausible extremes were retained in the primary analyses. Sensitivity analyses excluded exposure values below the 0.5th or above the 99.5th percentile. All Lp(a)-related models were repeated after removing extreme Lp(a) values, and separate datasets were created for SIRI, SII, and AISI using index-specific central 99% ranges. Original median cutoffs and covariate-adjustment strategies were retained.

To evaluate the robustness of the findings to the definition of hepatic steatosis, the primary logistic regression analyses were repeated using alternative CAP thresholds of 275, and 288 dB/m reported in previous validation studies and guidelines (28, 29). For the combined outcome, each alternative CAP threshold was applied together with LSM ≥8 kPa. To assess the robustness of the findings to the selection of the Lp(a) cutoff, sensitivity analyses were performed using thresholds of 170, 300, and 500 mg/L (30–32). For each analysis, participants with Lp(a) concentrations below the corresponding cutoff served as the reference group, and logistic regression analyses were repeated using the same covariate-adjustment strategy as in the primary analysis. Multivariable linear regression was used to evaluate the cross-sectional associations of Lp(a) with CAP and LSM as continuous outcomes. All statistical tests were two-sided, and a P value <0.05 was considered statistically significant.

## 3 Results

### 3.1 Baseline characteristics

Following the exclusion process, 1,345 participants were included in the final analysis. Lp(a) concentrations were categorized into quartiles, comprising Q1, Q2, Q3, and Q4, with corresponding sample sizes of 336, 336, 336, and 337, respectively. The baseline characteristics of the participants are presented in **Table 1**.

**Table 1:** Baseline characteristics of participants according to quartiles of Lp(a).

Variables	Total (n = 1345)	Q1 (n = 336)	Q2 (n = 336)	Q3 (n = 336)	Q4 (n = 337)	*P*
Age (years)	43.00 (29.00, 53.00)	45.00 (32.00,53.25)	43.00 (30.00,53.00)	43.50 (27.00,54.00)	42.00 (27.00,52.00)	0.085
Sex, n (%)						0.073
Male	698 (51.90)	184 (54.76)	188 (55.95)	167 (49.70)	159 (47.18)	
Female	647 (48.10)	152 (45.24)	148 (44.05)	169 (50.30)	178 (52.82)	
BMI (kg/m²)	25.67 ± 2.69	26.02 ± 2.70	25.81 ± 2.68	25.62 ± 2.67	25.24 ± 2.67	0.001
SBP (mmHg)	136.91 ± 12.04	138.01 ± 12.19	136.43 ± 12.05	137.07 ± 11.91	136.12 ± 11.99	0.182
DBP (mmHg)	84.97 ± 8.72	84.92 ± 8.77	84.59 ± 8.83	84.86 ± 8.52	85.50 ± 8.77	0.579
NAFLD, n (%)						<0.001
No	477 (35.46)	78 (23.21)	96 (28.57)	120 (35.71)	183 (54.30)	
Yes	868 (64.54)	258 (76.79)	240 (71.43)	216 (64.29)	154 (45.70)	
NAFLD with elevated liver stiffness, n (%)						<0.001
No	712 (52.94)	133 (39.58)	171 (50.89)	179 (53.27)	229 (67.95)	
Yes	633 (47.06)	203 (60.42)	165 (49.11)	157 (46.73)	108 (32.05)	
Hypertension, n (%)						0.314
No	562 (41.78)	131 (38.99)	147 (43.75)	133 (39.58)	151 (44.81)	
Yes	783 (58.22)	205 (61.01)	189 (56.25)	203 (60.42)	186 (55.19)	
DM, n (%)						0.415
0	636 (47.29)	152 (45.24)	172 (51.19)	156 (46.43)	156 (46.29)	
1	709 (52.71)	184 (54.76)	164 (48.81)	180 (53.57)	181 (53.71)	
WBC (×10^9^/L)	6.82 (5.65, 8.37)	6.95 (5.82,8.23)	6.83 (5.65,8.66)	6.95 (5.69,8.39)	6.59 (5.34,8.05)	0.045
NEU (×10^9^/L)	4.33 (3.35, 5.92)	4.43 (3.52,6.10)	4.33 (3.37,6.27)	4.39 (3.38,5.98)	4.22 (3.22,5.53)	0.053
LYM (×10^9^/L)	1.79 (1.41, 2.36)	1.79 (1.40,2.35)	1.83 (1.37,2.44)	1.82 (1.43,2.36)	1.75 (1.42,2.28)	0.878
MONO (×10^9^/L)	0.34 (0.25, 0.49)	0.36 (0.26,0.51)	0.33 (0.24,0.52)	0.34 (0.24,0.48)	0.34 (0.25,0.47)	0.386
PLT (×10^9^/L)	198.00 (150.35, 255.80)	198.00 (150.88,265.00)	200.62 (154.94,251.01)	184.00 (139.80,253.25)	206.00 (157.00,253.00)	0.184
SIRI	0.83 (0.54, 1.29)	0.90 (0.59,1.40)	0.81 (0.55,1.27)	0.81 (0.52,1.29)	0.80 (0.54,1.14)	0.144
SII	450.33 (327.65, 673.85)	460.92 (333.10,746.47)	442.04 (330.08,710.36)	433.03 (300.66,669.41)	455.89 (332.64,613.58)	0.286
AISI	162.86 (100.52, 267.62)	186.05 (104.45,299.18)	161.64 (99.45,272.33)	152.22 (92.98,255.05)	161.21 (105.19,254.43)	0.108
hs-CRP (mg/L)	4.32 (1.42, 15.01)	4.56 (1.58,16.57)	4.89 (1.28,17.01)	4.69 (1.74,12.97)	2.59 (1.20,12.89)	0.276
CAP (dB/m)	260.26 ± 35.57	271.74 ± 34.64	265.88 ± 36.04	258.31 ± 34.98	245.15 ± 30.87	<0.001
LSM (kPa)	7.84 ± 2.79	8.43 ± 2.84	8.07 ± 2.73	7.74 ± 2.82	7.12 ± 2.62	<0.001
TP (g/L)	68.19 ± 9.32	67.79 ± 9.15	67.76 ± 9.70	68.07 ± 9.72	69.14 ± 8.66	0.190
ALB (g/L)	41.69 ± 7.11	41.34 ± 6.75	41.58 ± 7.43	41.72 ± 7.29	42.10 ± 6.95	0.587
GLB (g/L)	26.05 ± 4.74	26.04 ± 4.97	25.72 ± 4.97	25.85 ± 4.69	26.59 ± 4.28	0.093
AGR (-)	1.63 ± 0.34	1.63 ± 0.34	1.65 ± 0.35	1.64 ± 0.33	1.62 ± 0.34	0.734
ALT (U/L)	57.30 (45.01, 69.00)	56.16 (42.95,70.34)	56.00 (42.15,68.03)	58.66 (46.21,68.85)	57.51 (47.18,68.75)	0.085
AST (U/L)	43.11 (34.00, 52.92)	42.86 (32.84,52.54)	40.39 (31.03,51.67)	44.52 (35.65,52.99)	44.43 (35.76,53.64)	<0.001
ALT/AST	1.31 (1.05, 1.60)	1.29 (1.02,1.62)	1.31 (1.01,1.64)	1.32 (1.07,1.60)	1.30 (1.07,1.55)	0.959
GGT (U/L)	39.00 (21.00, 81.73)	35.47 (20.00,69.75)	40.00 (21.00,78.50)	40.00 (21.00,76.25)	40.00 (22.50,100.00)	0.072
TBIL (μmol/L)	20.00 (13.74, 31.24)	19.97 (13.97,30.27)	20.45 (13.99,30.88)	19.60 (13.35,31.07)	19.60 (13.50,33.60)	0.796
DBIL (μmol/L)	5.20 (3.40, 9.80)	5.30 (3.50,9.50)	5.00 (3.40,9.28)	5.00 (3.10,9.65)	5.47 (3.40,11.31)	0.375
IBIL (μmol/L)	13.01 (8.90, 19.70)	13.00 (8.90,19.90)	13.25 (9.20,19.70)	13.06 (9.28,18.90)	12.80 (8.53,20.20)	0.835
TG (mmol/L)	1.82 (0.98, 2.42)	2.01 (1.18,2.61)	1.81 (0.91,2.42)	1.78 (0.95,2.38)	1.65 (0.98,2.32)	0.008
TC (mmol/L)	5.08 (3.78, 6.20)	5.24 (3.79,6.45)	5.09 (3.94,6.42)	5.05 (3.68,6.12)	4.92 (3.75,5.93)	0.053
HDL-C (mmol/L)	1.39 (1.05, 1.81)	1.41 (1.05,1.83)	1.36 (1.04,1.72)	1.44 (1.06,1.85)	1.36 (1.07,1.86)	0.349
LDL-C (mmol/L)	3.23 (2.46, 4.05)	3.24 (2.44,4.06)	3.28 (2.44,4.17)	3.08 (2.47,3.93)	3.26 (2.50,4.08)	0.578
Lp(a) (mg/L)	277 (192, 367)	144 (96,169)	237 (216,259)	320 (298,341)	423 (392,473)	<0.001
FPG (mmol/L)	6.22 (5.04, 8.23)	6.40 (5.10,8.46)	6.01 (4.91,7.84)	6.28 (5.20,8.40)	6.20 (4.98,8.30)	0.111
UA (μmol/L)	362.86 ± 96.90	371.80 ± 95.05	354.04 ± 94.26	360.52 ± 103.93	365.08 ± 93.57	0.111
eGFR (mL/min/1.73 m²)	85.45 ± 19.19	85.06 ± 20.71	85.56 ± 20.41	85.11 ± 18.30	86.06 ± 17.18	0.899

Data are presented as mean ± standard deviation, median (interquartile range), or number (percentage), as appropriate. Participants were grouped according to quartiles of Lp(a). Lp(a), lipoprotein(a); BMI, body mass index; SBP, systolic blood pressure; DBP, diastolic blood pressure; NAFLD, non-alcoholic fatty liver disease; DM, diabetes mellitus; WBC, white blood cell; NEU, neutrophil count; LYM, lymphocyte count; MONO, monocyte count; PLT, platelet count; SIRI, systemic inflammation response index; SII, systemic immune-inflammation index; AISI, aggregate index of systemic inflammation; hs-CRP, high-sensitivity C-reactive protein; CAP, controlled attenuation parameter; LSM, liver stiffness measurement; TP, total protein; ALB, albumin; GLB, globulin; AGR, albumin/globulin ratio; ALT, alanine aminotransferase; AST, aspartate aminotransferase; GGT, gamma-glutamyl transferase; TBIL, total bilirubin; DBIL, direct bilirubin; IBIL, indirect bilirubin; TG, triglycerides; TC, total cholesterol; HDL-C, high-density lipoprotein cholesterol; LDL-C, low-density lipoprotein cholesterol; FPG, fasting plasma glucose; UA, uric acid; eGFR, estimated glomerular filtration rate.

BMI values were progressively lower across increasing Lp(a) quartiles. The mean BMI values in Q1–Q4 were 26.02 ± 2.70, 25.81 ± 2.68, 25.62 ± 2.67, and 25.24 ± 2.67 kg/m², respectively, with a statistically significant difference among the groups (P = 0.001). The prevalence of NAFLD was also progressively lower across increasing Lp(a) quartiles, ranging from 76.79% in Q1 to 45.70% in Q4 (P < 0.001). A similar pattern was observed for prevalent NAFLD with elevated liver stiffness, with the corresponding proportion ranging from 60.42% in Q1 to 32.05% in Q4 (P < 0.001).

Participants in the higher Lp(a) quartiles had lower CAP and LSM values, with statistically significant differences among the groups for both measures (both P < 0.001). In Q4, the mean CAP and LSM values were 245.15 ± 30.87 dB/m and 7.12 ± 2.62 kPa, respectively. ALT and GGT did not differ significantly across Lp(a) quartiles, whereas AST differed significantly among the groups but showed no clear monotonic trend. TG levels were progressively lower across increasing Lp(a) quartiles (P = 0.008). WBC counts also differed among the groups (P = 0.045), with the lowest median value observed in Q4. No statistically significant differences across the Lp(a) quartiles were observed for age, sex, blood pressure, hypertension, diabetes, most liver function indicators, LDL-C, FPG, UA, or eGFR.

Additional outcome-based comparisons showed that Lp(a) concentrations were lower in participants with prevalent NAFLD than in those without NAFLD and, among participants with prevalent NAFLD, were lower in those with elevated liver stiffness than in those without elevated liver stiffness (**Supplementary Figure 2**; **Supplementary Tables 1**, **S2**).

### 3.2 Associations of Lp(a) with prevalent NAFLD and prevalent NAFLD with elevated liver stiffness

Before multivariable logistic regression, exploratory mediation and collinearity assessments were conducted for the laboratory variables included in Model 3. For prevalent NAFLD, none of the examined laboratory variables showed a statistically significant indirect effect in the association between Lp(a) and the outcome, and all bootstrap confidence intervals for the indirect effects included zero (**Supplementary Table 3**). Pairwise correlations between the selected laboratory variables and the systemic inflammatory indices were weak, with a maximum absolute correlation coefficient of 0.115 (**Supplementary Table 4**). Moreover, all VIF values were <5 and all tolerance values were >0.10, indicating no evidence of substantial multicollinearity among the examined variables (**Supplementary Table 5**).

Logistic regression showed that higher Lp(a) quartiles were associated with lower odds of prevalent NAFLD (**Table 2**). After adjustment for all included covariates and using Q1 as the reference category, the ORs were 0.70 for Q2 (95% CI, 0.48–1.03; P = 0.067), 0.53 for Q3 (95% CI, 0.37–0.76; P < 0.001), and 0.28 for Q4 (95% CI, 0.20–0.41; P < 0.001).

**Table 2:** Cross-sectional associations of Lp(a) with prevalent NAFLD and prevalent NAFLD with elevated liver stiffness.

Variables	Model 1	*P* value	Model 2	*P* value	Model 3	*P* value
NAFLD						
Continuous Lp(a)	0.63 (0.57 ~ 0.70)	**<0.001**	0.65 (0.59 ~ 0.72)	**<0.001**	0.66 (0.59 ~ 0.74)	**<0.001**
Lp(a) quartile						
Q1	Ref		Ref		Ref	
Q2	0.76 (0.53 ~ 1.07)	0.113	0.78 (0.55 ~ 1.11)	0.175	0.70 (0.48 ~ 1.03)	0.067
Q3	0.54 (0.39 ~ 0.76)	**<0.001**	0.57 (0.40 ~ 0.80)	**0.001**	0.53 (0.37 ~ 0.76)	**<0.001**
Q4	0.25 (0.18 ~ 0.35)	**<0.001**	0.28 (0.20 ~ 0.39)	**<0.001**	0.28 (0.20 ~ 0.41)	**<0.001**
NAFLD with elevated liver stiffness						
Continuous Lp(a)	0.70 (0.63 ~ 0.77)	**<0.001**	0.71 (0.64 ~ 0.78)	**<0.001**	0.70 (0.63 ~ 0.79)	**<0.001**
Lp(a) quartile						
Q1	Ref		Ref		Ref	
Q2	0.63 (0.47 ~ 0.86)	**0.003**	0.65 (0.48 ~ 0.89)	**0.008**	0.66 (0.46 ~ 0.94)	**0.023**
Q3	0.57 (0.42 ~ 0.78)	**<0.001**	0.59 (0.43 ~ 0.81)	**0.001**	0.65 (0.45 ~ 0.92)	**0.016**
Q4	0.31 (0.23 ~ 0.42)	**<0.001**	0.33 (0.24 ~ 0.46)	**<0.001**	0.31 (0.21 ~ 0.45)	**<0.001**

Multivariable logistic regression analyses were performed to evaluate the cross-sectional associations of Lp(a) with prevalent NAFLD and prevalent NAFLD with elevated liver stiffness. Lp(a) was analyzed both as a standardized continuous variable and by quartiles. For continuous analyses, odds ratios represent the change in the odds of the prevalent outcome per 1-standard-deviation increase in Lp(a). Q1 was used as the reference category. Data are presented as odds ratios with 95% confidence intervals. Model 1 was unadjusted. Model 2 was adjusted for age, sex, hypertension, and BMI. Model 3 was further adjusted for diabetes mellitus, ALT, AST, TC, TG, HDL-C, LDL-C, UA, eGFR, GGT, ALB, GLB, DBIL, and IBIL. All analyses were based on participants with complete data for the variables included in the corresponding model.

OR, odds ratio; CI, confidence interval.

Bold values indicate statistical significance (P < 0.05).

A similar inverse association was observed for prevalent NAFLD with elevated liver stiffness. When modeled continuously, higher Lp(a) levels were consistently associated with lower odds of the outcome across all regression models. In the fully adjusted quartile analysis, the ORs were 0.66 for Q2 (95% CI, 0.46–0.94; P = 0.023), 0.65 for Q3 (95% CI, 0.45–0.92; P = 0.016), and 0.31 for Q4 (95% CI, 0.21–0.45; P < 0.001), relative to Q1.

**Figure 2:**
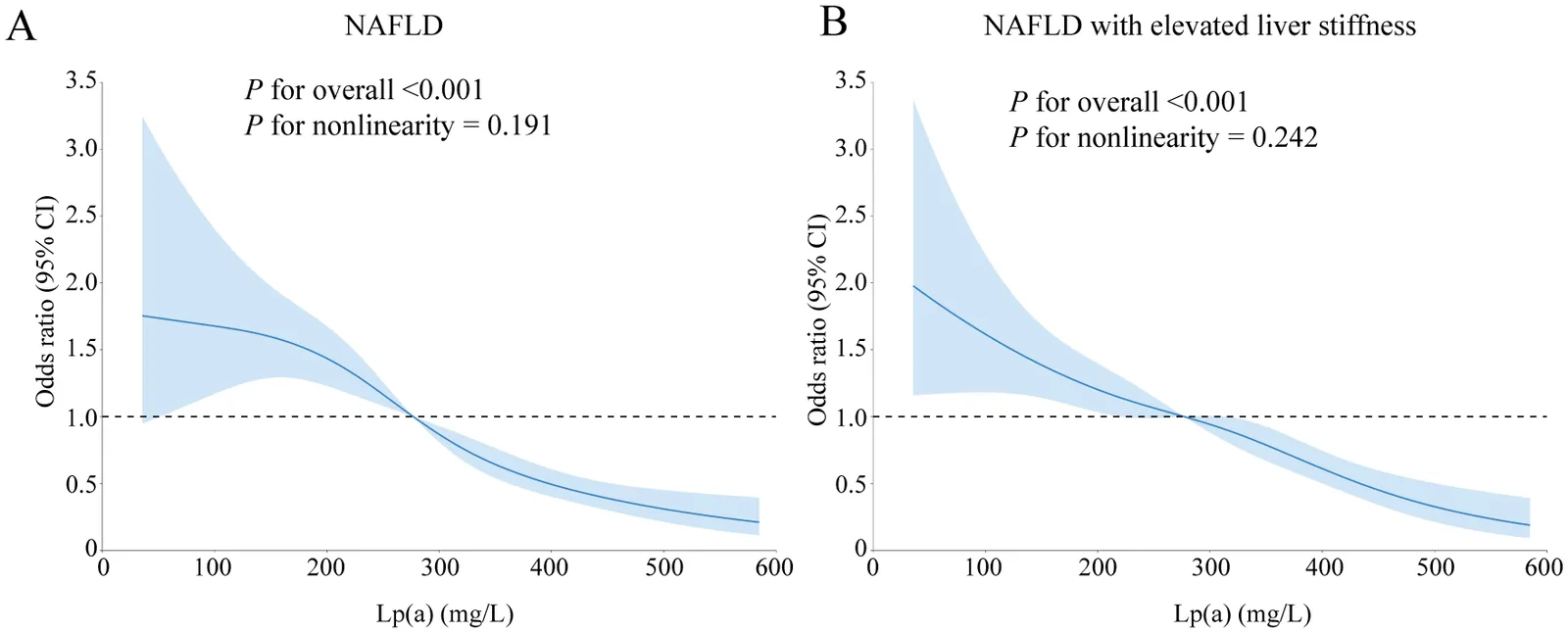
RCS analyses of the cross-sectional associations of Lp(a) with prevalent NAFLD and prevalent NAFLD with elevated liver stiffness. **(a)** Association of Lp(a) with prevalent NAFLD. **(b)** Association of Lp(a) with prevalent NAFLD with elevated liver stiffness.

The shape of the cross-sectional associations was further examined using restricted cubic spline analysis (**Figure 2**). Covariate adjustment was consistent with Model 3 in **Table 2**. RCS analyses further demonstrated significant overall inverse associations of Lp(a) with prevalent NAFLD and prevalent NAFLD with elevated liver stiffness (both P for overall < 0.001). No statistically significant evidence of nonlinearity was observed for either prevalent NAFLD (P for nonlinearity = 0.191) or prevalent NAFLD with elevated liver stiffness (P for nonlinearity = 0.242), indicating approximately linear inverse associations (**Figure 2**).

### 3.3 Joint-exposure analysis

In the joint-exposure analyses, Lp(a), SIRI, SII, and AISI were dichotomized at their respective medians. Values below the median were classified as low and those at or above the median as high. The high-Lp(a)/low-inflammation group, expected to have the lowest odds based on the individual associations, served as the reference. Both lower Lp(a) concentrations and higher inflammatory index levels were associated with higher odds of prevalent NAFLD and prevalent NAFLD with elevated liver stiffness.

For prevalent NAFLD, participants with low Lp(a) and low inflammation, as well as those with high Lp(a) and high inflammation, generally had higher odds than the reference category. The highest odds were observed among participants with both low Lp(a) and a high inflammatory burden. In the adjusted models, the ORs for prevalent NAFLD were 3.63 for low Lp(a) combined with high SIRI (95% CI, 2.51–5.23), 4.23 for low Lp(a) combined with high SII (95% CI, 2.90–6.16), and 3.15 for low Lp(a) combined with high AISI (95% CI, 2.20–4.52) (**Table 3**).

**Table 3:** Joint associations of Lp(a) and systemic inflammatory index categories with prevalent NAFLD and prevalent NAFLD with elevated liver stiffness.

Outcome	Joint exposure group	n	Events	Adjusted OR (95% CI)	P value
NAFLD	High Lp(a) + low SIRI	319	162	1.00 (Reference)	
	Low Lp(a) + low SIRI	282	189	1.90 (1.35-2.68)	<0.001
	High Lp(a) + high SIRI	284	168	1.48 (1.05-2.07)	0.024
	Low Lp(a) + high SIRI	320	257	3.63 (2.51-5.23)	<0.001
	High Lp(a) + low SII	303	142	1.00 (Reference)	
	Low Lp(a) + low SII	299	202	2.11 (1.50-2.98)	<0.001
	High Lp(a) + high SII	300	188	1.82 (1.30-2.56)	<0.001
	Low Lp(a) + high SII	303	244	4.23 (2.90-6.16)	<0.001
	High Lp(a) + low AISI	317	161	1.00 (Reference)	
	Low Lp(a) + low AISI	285	197	2.01 (1.42-2.84)	<0.001
	High Lp(a) + high AISI	286	169	1.37 (0.98-1.92)	0.065
	Low Lp(a) + high AISI	317	249	3.15 (2.20-4.52)	<0.001
NAFLD with elevated liver stiffness	High Lp(a) + low SIRI	319	104	1.00 (Reference)	
	Low Lp(a) + low SIRI	282	136	1.96 (1.37-2.82)	<0.001
	High Lp(a) + high SIRI	284	136	1.82 (1.26-2.61)	0.001
	Low Lp(a) + high SIRI	320	187	2.80 (1.96-4.00)	<0.001
	High Lp(a) + low SII	303	112	1.00 (Reference)	
	Low Lp(a) + low SII	299	145	1.52 (1.06-2.18)	0.022
	High Lp(a) + high SII	300	128	1.06 (0.74-1.52)	0.759
	Low Lp(a) + high SII	303	178	2.20 (1.53-3.17)	<0.001
	High Lp(a) + low AISI	317	105	1.00 (Reference)	
	Low Lp(a) + low AISI	285	140	2.01 (1.40-2.89)	<0.001
	High Lp(a) + high AISI	286	135	1.70 (1.18-2.43)	0.004
	Low Lp(a) + high AISI	317	183	2.59 (1.81-3.70)	<0.001

Multivariable logistic regression analyses were performed to evaluate the joint cross-sectional associations of Lp(a) and systemic inflammatory indices with prevalent NAFLD and prevalent NAFLD with elevated liver stiffness. Lp(a), SIRI, SII, and AISI were dichotomized according to their respective median values: 277 mg/L for Lp(a), 0.83 for SIRI, 450.33 for SII, and 162.86 for AISI. Values below the median were classified as low, whereas values equal to or above the median were classified as high. The high-Lp(a)/low-inflammation category was used as the reference category. Data are presented as adjusted odds ratios with 95% confidence intervals. The models were adjusted for the same covariates as Model 3 in **Table 2**.

A similar pattern was observed for prevalent NAFLD with elevated liver stiffness. Participants with low Lp(a) combined with elevated inflammatory index levels consistently had the highest odds of the outcome. In the adjusted joint-exposure models, the corresponding ORs were 2.80 for low Lp(a) combined with high SIRI (95% CI, 1.96–4.00), 2.20 for low Lp(a) combined with high SII (95% CI, 1.53–3.17), and 2.59 for low Lp(a) combined with high AISI (95% CI, 1.81–3.70) (**Table 3**).

No statistically significant multiplicative interactions were observed between Lp(a) and SIRI, SII, or AISI for either prevalent NAFLD or prevalent NAFLD with elevated liver stiffness. On the additive scale, neither relative excess risk due to interaction (RERI) nor synergy index (SI) was statistically significant for any of the inflammatory indices. Although the attributable proportion for the Lp(a) × SIRI combination reached statistical significance in the NAFLD analysis, this finding was not accompanied by statistically significant RERI or SI estimates. Taken together, these results did not provide consistent evidence of either multiplicative or additive interaction between Lp(a) and the systemic inflammatory indices examined in this study (**Table 4**).

**Table 4:** Multiplicative and additive interaction measures between Lp(a) and systemic inflammatory indices for prevalent NAFLD and prevalent NAFLD with elevated liver stiffness.

Outcome	Interaction pair	Measures	Estimates (95% CI)	P-value
NAFLD	Lp(a) × SIRI	Multiplicative interaction	1.29 (0.70, 2.37)	0.409
		RERI (95% CI)	1.25 (-0.32, 2.82)	0.118
		AP (95% CI)	0.34 (0.01, 0.67)	0.041
		SI (95% CI)	1.91 (0.87, 4.17)	0.106
	Lp(a) × SII	Multiplicative interaction	1.10 (0.60, 2.03)	0.757
		RERI (95% CI)	1.30 (-0.56, 3.16)	0.170
		AP (95% CI)	0.31 (-0.04, 0.65)	0.080
		SI (95% CI)	1.67 (0.83, 3.36)	0.148
	Lp(a) × AISI	Multiplicative interaction	1.14 (0.63, 2.09)	0.662
		RERI (95% CI)	0.77 (-0.64, 2.18)	0.284
		AP (95% CI)	0.24 (-0.14, 0.62)	0.207
		SI (95% CI)	1.56 (0.70, 3.48)	0.279
NAFLD with elevated liver stiffness	Lp(a) × SIRI	Multiplicative interaction	0.78 (0.42, 1.47)	0.447
		RERI (95% CI)	0.02 (-1.37, 1.41)	0.978
		AP (95% CI)	0.01 (-0.49, 0.50)	0.977
		SI (95% CI)	1.01 (0.46, 2.20)	0.978
	Lp(a) × SII	Multiplicative interaction	1.37 (0.73, 2.55)	0.330
		RERI (95% CI)	0.62 (-0.42, 1.66)	0.244
		AP (95% CI)	0.28 (-0.12, 0.68)	0.168
		SI (95% CI)	2.07 (0.55, 7.83)	0.284
	Lp(a) × AISI	Multiplicative interaction	0.76 (0.41, 1.41)	0.384
		RERI (95% CI)	-0.12 (-1.45, 1.21)	0.859
		AP (95% CI)	-0.05 (-0.57, 0.48)	0.863
		SI (95% CI)	0.93 (0.42, 2.08)	0.860

Interaction analyses were performed to evaluate potential statistical interactions between Lp(a) and SIRI, SII, or AISI in relation to prevalent NAFLD and prevalent NAFLD with elevated liver stiffness. Multiplicative interaction was assessed by including a cross-product term in the multivariable logistic regression model. Additive interaction was evaluated using RERI, AP, and SI. Values of RERI >0, AP >0, and SI >1 indicate positive additive interaction, whereas RERI = 0, AP = 0, and SI = 1 indicate no additive interaction. Estimates are presented with 95% confidence intervals. Models were adjusted for age, sex, hypertension, diabetes mellitus, BMI, ALT, AST, TC, TG, HDL-C, LDL-C, UA, eGFR, GGT, ALB, GLB, DBIL, and IBIL.

### 3.4 Discriminatory performance of Lp(a), inflammatory indices, and combined models

ROC curve analyses showed that the individual biomarkers had modest discriminatory performance. For discriminating participants with and without prevalent NAFLD, Lp(a) had the largest AUC among the individual markers, with an AUC of 0.650 (95% CI, 0.619–0.681). SII had the next largest AUC, at 0.643 (95% CI, 0.614–0.672), whereas SIRI and AISI showed comparatively lower discriminatory ability.

**Figure 3:**
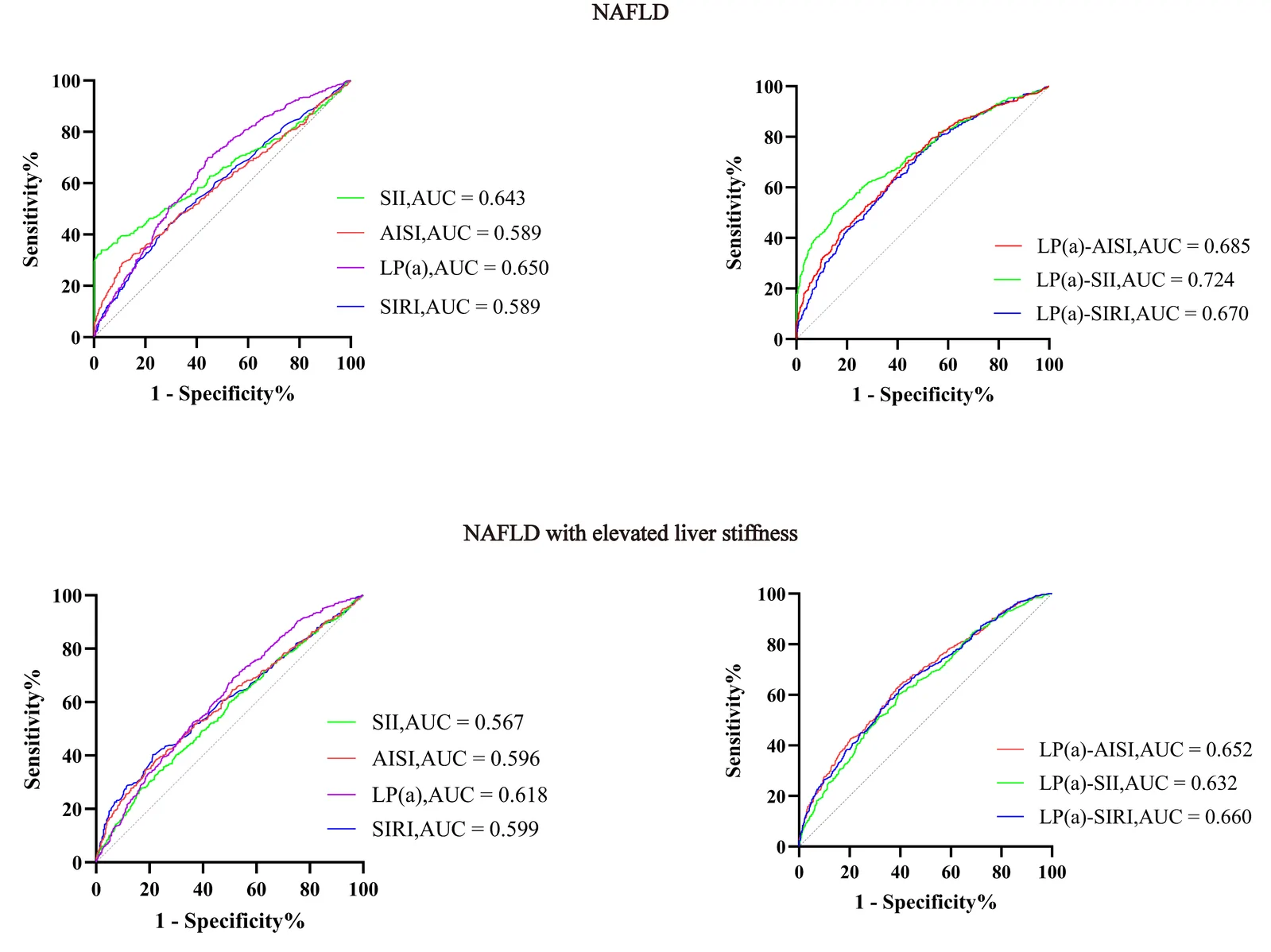
Receiver operating characteristic curves for discriminating prevalent NAFLD and prevalent NAFLD with elevated liver stiffness using Lp(a), systemic inflammatory indices, and their combined models.

Within the study sample, the biomarker-only models combining Lp(a) with inflammatory indices yielded higher AUCs than the individual markers. Among these models, Lp(a) plus SII showed the highest AUC for prevalent NAFLD, with an AUC of 0.724 (95% CI, 0.698–0.751). For prevalent NAFLD with elevated liver stiffness, Lp(a) plus SIRI showed the highest AUC, at 0.660 (95% CI, 0.631–0.689) (**Figure 3**, **Table 5**). These findings indicate that the biomarker-only combinations provided greater discriminatory information than the individual markers, although their overall discriminatory ability remained moderate.

**Table 5:** Discriminatory performance of Lp(a), systemic inflammatory indices, and their combined models for prevalent NAFLD and prevalent NAFLD with elevated liver stiffness.

Variables	AUC (95% CI)	Accuracy (95% CI)	Sensitivity (95% CI)	Specificity (95% CI)	PPV (95% CI)	NPV (95% CI)	Cut off
NAFLD							
Lp(a)	0.650 (0.619-0.681)	0.352 (0.326-0.378)	0.442 (0.398 - 0.487)	0.302 (0.271 - 0.332)	0.258 (0.228 - 0.288)	0.496 (0.454 - 0.539)	314.835
SIRI	0.589 (0.557-0.619)	0.535 (0.507-0.562)	0.709 (0.668 - 0.749)	0.439 (0.406 - 0.472)	0.410 (0.376 - 0.443)	0.733 (0.695 - 0.771)	1.005
SII	0.643 (0.614-0.672)	0.564 (0.537-0.590)	0.971 (0.956 - 0.986)	0.340 (0.308 - 0.371)	0.447 (0.417 - 0.477)	0.955 (0.932 - 0.978)	709.305
AISI	0.589 (0.559-0.620)	0.503 (0.476-0.530)	0.887 (0.858 - 0.915)	0.291 (0.261 - 0.322)	0.408 (0.378 - 0.437)	0.824 (0.782 - 0.867)	288.200
Lp(a)-SIRI	0.670 (0.640-0.700)	0.653 (0.627-0.678)	0.522 (0.477 - 0.567)	0.725 (0.695 - 0.754)	0.510 (0.466 - 0.555)	0.734 (0.704 - 0.764)	0.599
Lp(a)-SII	0.724 (0.698-0.751)	0.623 (0.597-0.649)	0.851 (0.819 - 0.883)	0.498 (0.464 - 0.531)	0.482 (0.448 - 0.516)	0.859 (0.828 - 0.889)	0.714
Lp(a)-AISI	0.685 (0.656-0.714)	0.653 (0.627-0.678)	0.556 (0.511 - 0.600)	0.706 (0.676 - 0.737)	0.510 (0.467 - 0.553)	0.743 (0.713 - 0.773)	0.599
NAFLD with elevated liver stiffness							
Lp(a)	0.618 (0.588-0.647)	0.418 (0.391-0.445)	0.517 (0.480 - 0.554)	0.306 (0.271 - 0.342)	0.456 (0.422 - 0.490)	0.361 (0.320 - 0.401)	311.915
SIRI	0.599 (0.569-0.630)	0.607 (0.580-0.633)	0.788 (0.758 - 0.818)	0.403 (0.365 - 0.441)	0.597 (0.566 - 0.629)	0.628 (0.581 - 0.675)	1.155
SII	0.567 (0.536-0.598)	0.570 (0.543-0.597)	0.830 (0.802 - 0.858)	0.278 (0.243 - 0.313)	0.564 (0.534 - 0.594)	0.593 (0.537 - 0.648)	716.07
AISI	0.596 (0.566-0.627)	0.592 (0.565-0.618)	0.775 (0.745 - 0.806)	0.385 (0.348 - 0.423)	0.587 (0.555 - 0.618)	0.604 (0.556 - 0.652)	243.645
Lp(a)-SIRI	0.660 (0.631-0.689)	0.621 (0.594-0.647)	0.618 (0.582 - 0.654)	0.624 (0.586 - 0.662)	0.649 (0.613 - 0.685)	0.592 (0.555 - 0.629)	0.457
Lp(a)-SII	0.632 (0.603-0.662)	0.604 (0.578-0.631)	0.610 (0.574 - 0.645)	0.599 (0.561 - 0.637)	0.631 (0.595 - 0.667)	0.577 (0.539 - 0.615)	0.469
Lp(a)-AISI	0.652 (0.623-0.681)	0.611 (0.585-0.637)	0.601 (0.565 - 0.637)	0.622 (0.585 - 0.660)	0.642 (0.605 - 0.678)	0.581 (0.544 - 0.618)	0.452

Receiver operating characteristic curve analyses were performed to evaluate the discriminative performance of Lp(a), SIRI, SII, AISI, and their combined indices for NAFLD and NAFLD with elevated liver stiffness. Data are presented as AUC, accuracy, sensitivity, specificity, PPV, and NPV with 95% confidence intervals. The optimal cutoff value was determined according to the maximum Youden index.AUC, area under the receiver operating characteristic curve; CI, confidence interval; PPV, positive predictive value; NPV, negative predictive value.

When the biomarkers were added to the fully adjusted Model 3, greater discriminatory performance was observed. For prevalent NAFLD, adding Lp(a) plus SII yielded the largest AUC of 0.762 (95% CI, 0.735–0.789), compared with 0.717 for Lp(a) plus SIRI and 0.727 for Lp(a) plus AISI. The corresponding AUCs after adding FIB-4, APRI, and NFS were 0.664, 0.654, and 0.665, respectively (**Supplementary Table 6**).

For prevalent NAFLD with elevated liver stiffness, adding Lp(a) plus SIRI to Model 3 yielded the largest AUC of 0.811 (95% CI, 0.781–0.840), followed by Lp(a) plus AISI with an AUC of 0.809 (95% CI, 0.779–0.839) and Lp(a) plus SII with an AUC of 0.799 (95% CI, 0.768–0.830). The corresponding AUCs after adding FIB-4, APRI, and NFS were 0.760, 0.751, and 0.762, respectively (**Supplementary Table 7**).

IDI and NRI analyses further evaluated incremental discrimination and reclassification beyond the fully adjusted base model. For prevalent NAFLD, adding Lp(a) plus SII produced the greatest improvement (IDI, 11.80%; NRI, 59.56%), whereas Lp(a) plus SIRI and Lp(a) plus AISI also improved performance; FIB-4, APRI, and NFS showed smaller gains (**Supplementary Table 8**). For prevalent NAFLD with elevated liver stiffness, Lp(a) plus SIRI yielded the greatest improvement (IDI, 8.20%; NRI, 56.16%), followed closely by Lp(a) plus AISI, while FIB-4, APRI, and NFS showed more limited and less consistent improvements (**Supplementary Table 9**).

### 3.5 Subgroup analysis

The consistency of the cross-sectional associations between Lp(a) and the liver-related outcomes was examined in stratified analyses according to demographic and clinical characteristics, including age, sex, BMI, hypertension, diabetes, and other selected variables. The subgroup-specific results are presented in the forest plots.

**Figure 4:**
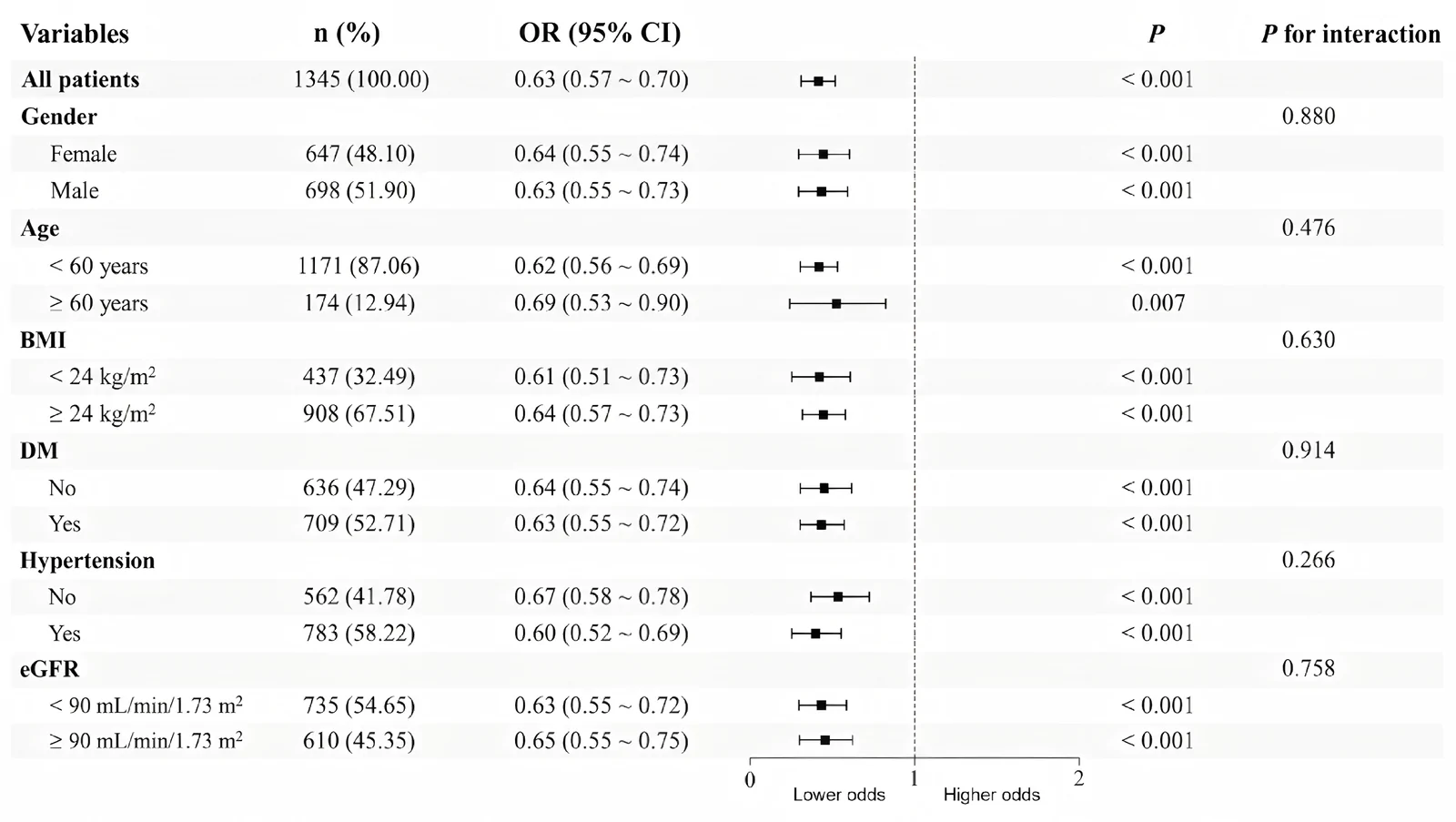
Subgroup analyses of the cross-sectional association between Lp(a) and prevalent NAFLD.

**Figure 5:**
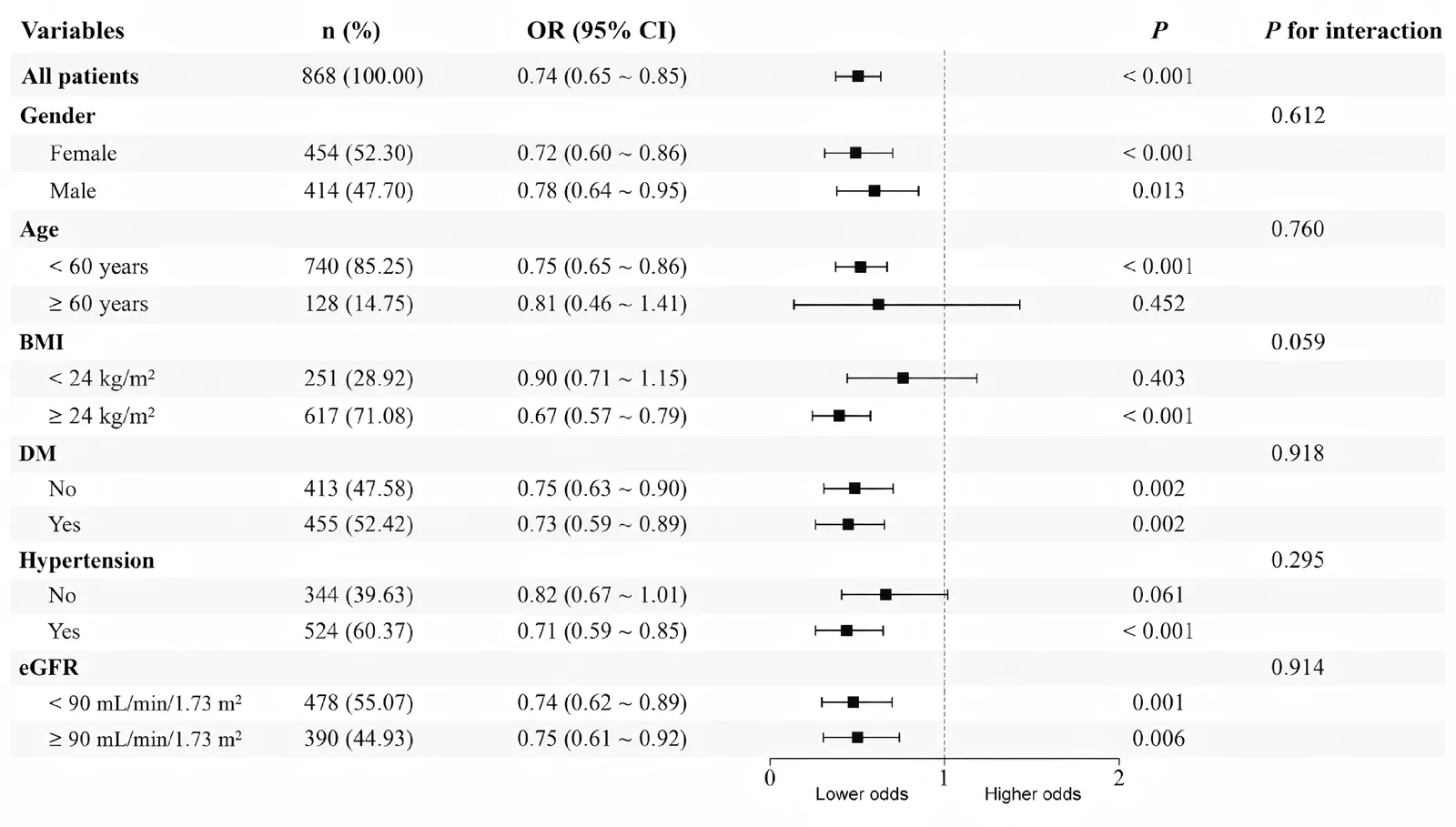
Subgroup analyses of the cross-sectional association between Lp(a) and prevalent NAFLD with elevated liver stiffness.

In most subgroups, higher Lp(a) levels were associated with lower odds of prevalent NAFLD, with most ORs below 1. No substantial heterogeneity was observed across demographic and metabolic strata, suggesting that the inverse association was generally consistent across the examined subgroups (**Figure 4**). A similar pattern was observed for prevalent NAFLD with elevated liver stiffness, for which most subgroup-specific ORs were also below 1 (**Figure 5**).

### 3.6 Sensitivity analysis

Sensitivity analyses were conducted using Lp(a) cutoff values of 170, 300, and 500 mg/L. After full adjustment, Lp(a) ≥170 mg/L was inversely associated with prevalent NAFLD (OR, 0.42; 95% CI, 0.29–0.60; P <0.001) and prevalent NAFLD with elevated liver stiffness (OR, 0.53; 95% CI, 0.38–0.73; P <0.001). Similar inverse associations were observed using the 300 mg/L cutoff, with corresponding ORs of 0.46 (95% CI, 0.36–0.60; P <0.001) and 0.54 (95% CI, 0.42–0.69; P <0.001), respectively. At the 500 mg/L cutoff, Lp(a) ≥500 mg/L was inversely associated with prevalent NAFLD with elevated liver stiffness (OR, 0.25; 95% CI, 0.11–0.59; P = 0.002) (**Table 6**). Overall, the sensitivity analyses supported the robustness of the inverse associations across alternative Lp(a) cutoff values. Additional sensitivity analyses using alternative CAP thresholds of 275, and 288 dB/m showed that the inverse associations of Lp(a) ≥300 mg/L with prevalent NAFLD and prevalent NAFLD with elevated liver stiffness were generally consistent with the primary findings (**Supplementary Tables 10**, **S11**).

**Table 6:** Sensitivity analyses of the cross-sectional associations of Lp(a) with prevalent NAFLD and prevalent NAFLD with elevated liver stiffness using alternative cutoff values.

Lp(a) cutoff	Model	Outcome	n	Events, n	Event rate, %	OR (95% CI)	P value
≥170 mg/L	Model 1	NAFLD	1345	868	64.5	0.46 (0.34–0.64)	<0.001
		NAFLD with elevated liver stiffness	1345	633	47.1	0.50 (0.38–0.66)	<0.001
	Model 2	NAFLD	1345	868	64.5	0.49 (0.35–0.67)	<0.001
		NAFLD with elevated liver stiffness	1345	633	47.1	0.51 (0.39–0.69)	<0.001
	Model 3	NAFLD	1205	776	64.4	0.42 (0.29–0.60)	<0.001
		NAFLD with elevated liver stiffness	1205	563	46.7	0.53 (0.38–0.73)	<0.001
≥300 mg/L	Model 1	NAFLD	1345	868	64.5	0.43 (0.34–0.55)	<0.001
		NAFLD with elevated liver stiffness	1345	633	47.1	0.53 (0.43–0.66)	<0.001
	Model 2	NAFLD	1345	868	64.5	0.44 (0.35–0.55)	<0.001
		NAFLD with elevated liver stiffness	1345	633	47.1	0.53 (0.43–0.67)	<0.001
	Model 3	NAFLD	1205	776	64.4	0.46 (0.36–0.60)	<0.001
		NAFLD with elevated liver stiffness	1205	563	46.7	0.54 (0.42–0.69)	<0.001
≥500 mg/L	Model 1	NAFLD	1345	868	64.5	0.36 (0.20–0.65)	<0.001
		NAFLD with elevated liver stiffness	1345	633	47.1	0.33 (0.17–0.66)	0.002
	Model 2	NAFLD	1345	868	64.5	0.37 (0.20–0.68)	0.001
		NAFLD with elevated liver stiffness	1345	633	47.1	0.33 (0.16–0.67)	0.002
	Model 3	NAFLD	1205	776	64.4	0.54 (0.28–1.04)	0.064
		NAFLD with elevated liver stiffness	1205	563	46.7	0.25 (0.11–0.59)	0.002

Lp(a) concentrations were categorized using cutoff values of 170, 300, and 500 mg/L, with values below each respective cutoff serving as the reference. Model 1 was unadjusted. Model 2 was adjusted for age, sex, hypertension, and BMI. Model 3 was further adjusted for diabetes mellitus, ALT, AST, TC, TG, HDL-C, LDL-C, UA, eGFR, GGT, ALB, GLB, DBIL, and IBIL. The sample size in Model 3 was reduced to 1,205 because the fully adjusted analyses were restricted to participants with complete data for all included covariates. OR, odds ratio; CI, confidence interval; NAFLD, nonalcoholic fatty liver disease; Lp(a), lipoprotein(a).

Exclusion of extreme Lp(a) values did not materially alter the findings. Lp(a) remained inversely associated with both outcomes in continuous, quartile, joint-exposure, and threshold-based analyses, and low Lp(a) combined with high inflammation continued to confer the highest odds (**Supplementary Tables 12**-**S14**). Similarly, after trimming extreme inflammatory-index values, SIRI and AISI remained positively associated with both outcomes, and SII remained positively associated with prevalent NAFLD after full adjustment (**Supplementary Table 15**).

Multiple-imputation analyses among 2,349 participants yielded results consistent with the complete-case analyses. Elevated Lp(a) was inversely associated with prevalent NAFLD across all three models. In the fully adjusted model, Lp(a) ≥300 mg/L remained significantly associated with lower odds of NAFLD compared with Lp(a) <300 mg/L (OR, 0.50; 95% CI, 0.41–0.62; P <0.001), supporting the robustness of the association to the handling of missing covariate data (**Supplementary Table 16**).

### 3.7 Associations of Lp(a) with CAP and LSM as continuous variables

When CAP and LSM were analyzed as continuous outcomes, higher Lp(a) levels were associated with lower CAP values. In the fully adjusted analysis, higher continuous Lp(a) levels remained inversely associated with CAP, with a β estimate of −14.40 (95% CI, −17.79 to −11.01; P < 0.001). Compared with Q1, participants in Q2, Q3, and Q4 had lower CAP values, with the largest negative β estimate observed in Q4 (β = −22.62; 95% CI, −28.04 to −17.20; P < 0.001).

Lp(a) was also inversely associated with LSM. After full covariate adjustment, higher continuous Lp(a) concentrations were associated with lower LSM values, with a β estimate of −0.58 (95% CI, −0.83 to −0.33; P < 0.001). In the quartile analyses, participants in Q3 (β = −0.40; 95% CI, −0.79 to −0.01; P = 0.046) and Q4 (β = −0.96; 95% CI, −1.36 to −0.56; P < 0.001) had lower LSM values than those in Q1, whereas no statistically significant difference was observed between Q2 and Q1 (**Table 7**).

**Table 7:** Multivariable linear regression associations of Lp(a) with CAP and LSM as continuous outcomes.

Variables	Model 1		Model 2		Model 3	
	β (95% CI)	*P* value	β (95% CI)	*P* value	β (95% CI)	*P* value
CAP						
Continuous Lp(a)	-16.21 (-19.42 ~ -12.99)	<0.001	-15.16 (-18.37 ~ -11.95)	<0.001	-14.40 (-17.79 ~ -11.01)	<0.001
Lp(a) quartile						
Q1	Ref		Ref		Ref	
Q2	-5.86 (-11.03 ~ -0.69)	0.026	-5.32 (-10.45 ~ -0.19)	0.042	-5.84 (-11.20 ~ -0.48)	0.033
Q3	-13.43 (-18.60 ~ -8.26)	<0.001	-12.54 (-17.68 ~ -7.40)	<0.001	-12.34 (-17.67 ~ -7.01)	<0.001
Q4	-26.59 (-31.76 ~ -21.43)	<0.001	-24.85 (-30.02 ~ -19.68)	<0.001	-22.62 (-28.04 ~ -17.20)	<0.001
LSM						
Continuous Lp(a)	-0.80 (-1.05 ~ -0.54)	<0.001	-0.69 (-0.94 ~ -0.44)	<0.001	-0.58 (-0.83 ~ -0.33)	<0.001
Lp(a) quartile						
Q1	Ref		Ref		Ref	
Q2	-0.36 (-0.77 ~ 0.06)	0.092	-0.27 (-0.68 ~ 0.13)	0.182	-0.15 (-0.54 ~ 0.24)	0.455
Q3	-0.69 (-1.10 ~ -0.27)	0.001	-0.58 (-0.98 ~ -0.18)	0.005	-0.40 (-0.79 ~ -0.01)	0.046
Q4	-1.30 (-1.72 ~ -0.89)	<0.001	-1.09 (-1.49 ~ -0.68)	<0.001	-0.96 (-1.36 ~ -0.56)	<0.001

Multivariable linear regression analyses were performed to evaluate the cross-sectional associations of Lp(a) with CAP and LSM as continuous outcomes. Lp(a) was analyzed both as a standardized continuous variable and by quartiles. For continuous analyses, β coefficients represent the mean difference in CAP or LSM per 1-standard-deviation increase in Lp(a). For the quartile analyses, Q1 was used as the reference category. β coefficients for CAP are expressed in dB/m, and β coefficients for LSM are expressed in kPa. Data are presented as β coefficients with 95% confidence intervals. Model 1 was unadjusted. Model 2 was adjusted for age, sex, hypertension, and BMI. Model 3 was further adjusted for diabetes mellitus, ALT, AST, TC, TG, HDL-C, LDL-C, UA, eGFR, GGT, ALB, GLB, DBIL, and IBIL.

## 4 Discussion

Using data from 1,345 adults recruited in a Chinese hospital setting, this study evaluated the cross-sectional associations of Lp(a), blood cell-derived inflammatory indices, and combined lipid–inflammatory models with prevalent NAFLD, NAFLD with elevated liver stiffness, CAP, and LSM. Higher Lp(a) concentrations were inversely associated with both prevalent outcomes after multivariable adjustment, with restricted cubic spline analyses showing no evidence of nonlinearity. Lp(a) was also inversely associated with CAP and LSM. Although individual biomarkers showed modest discrimination, combinations of Lp(a) with inflammatory indices yielded numerically higher AUCs, with Lp(a)–SII performing best for prevalent NAFLD and Lp(a)–SIRI for NAFLD with elevated liver stiffness. Participants with low Lp(a) and high inflammatory burden had the highest odds of both outcomes. The positive IDI and NRI estimates further suggested that lipid- and inflammation-related biomarkers may provide complementary information beyond conventional clinical variables.

NAFLD develops through interacting metabolic, oxidative, mitochondrial, and inflammatory processes. The multiple-hit hypothesis emphasizes the combined roles of insulin resistance, hepatic lipid accumulation, oxidative injury, gut-derived signals, and inflammation (33). Inflammatory activation may occur early during the progression from steatosis to steatohepatitis and fibrotic remodeling (34, 35). These mechanisms provide a biological rationale for evaluating systemic inflammatory indices in relation to hepatic steatosis and stiffness. In the present study, inflammatory indices alone showed limited-to-moderate discrimination, whereas their combination with Lp(a) provided greater cross-sectional discriminatory information.

Lp(a) is a largely genetically determined causal risk factor for atherosclerotic cardiovascular disease, but apo(a) is synthesized primarily by hepatocytes. Circulating Lp(a) concentrations may therefore also be influenced by hepatic metabolic and synthetic alterations. Previous studies have reported lower Lp(a) concentrations in individuals with NAFLD, NASH, and advanced liver fibrosis (7, 36–39). More recent biopsy-based and population-based studies have similarly shown inverse associations of Lp(a) with at-risk MASH, MASLD, and liver fat content (40, 41). These findings support the direction of our results, although sex, hormonal status, disease severity, and study design may modify the observed associations.

Several mechanisms may contribute to lower Lp(a) concentrations in individuals with more severe liver abnormalities. Progressive steatosis and fibrotic remodeling may alter hepatocellular lipoprotein synthesis and secretion. Meroni et al. reported that hepatic LPA expression was positively correlated with circulating Lp(a), and that both decreased with increasing NAFLD severity (37). Insulin resistance, hyperinsulinemia, oxidative stress, inflammation, and mitochondrial dysfunction may also influence apo(a) production or Lp(a) metabolism. However, current evidence is predominantly observational, and substantial between-study heterogeneity remains (42). Lower Lp(a) should therefore not be interpreted as hepatoprotective or as evidence of reduced cardiovascular risk. Reverse causality is possible, whereby hepatic metabolic or synthetic dysfunction lowers circulating Lp(a). Moreover, MASLD itself increases cardiovascular risk through metabolic dysfunction, inflammation, endothelial injury, and liver–heart crosstalk (43). Because LPA genotype, apo(a) isoform size, insulin levels, and hepatic LPA expression were not assessed, the proposed mechanisms remain speculative.

SII, SIRI, and AISI integrate information from neutrophils, lymphocytes, monocytes, and platelets and can be calculated from routine blood counts (23–25, 44, 45). Previous studies have associated these indices with hepatic steatosis, fibrosis-related measures, and clinical outcomes (46–48). In the present study, Lp(a)–SII had the highest AUC for prevalent NAFLD, whereas Lp(a)–SIRI performed best for NAFLD with elevated liver stiffness. This difference may reflect the distinct cellular components represented by SII and SIRI, but it does not establish the involvement of specific inflammatory pathways. Further mechanistic and longitudinal studies are required.

Lp(a) and systemic inflammatory indices are routinely available and inexpensive to derive. Their combination may provide a broader cross-sectional characterization of hepatic lipid metabolism, systemic inflammation, and stiffness-related abnormalities than either biomarker domain alone. Nevertheless, the observed associations do not establish prospective predictive value. The biomarker-only models showed only moderate discrimination, and the multivariable models were developed and evaluated within the same study sample. Therefore, internal validation, external validation, and prospective studies are needed before these combinations can be used to guide liver imaging, metabolic assessment, or cardiovascular evaluation.

This study has several strengths. CAP and LSM provided quantitative noninvasive measures of hepatic fat accumulation and liver stiffness. Lp(a) was analyzed in relation to both dichotomous outcomes and continuous CAP and LSM values, and the generally consistent findings across these approaches supported the robustness of the observed associations. The study also incorporated joint-exposure, subgroup, sensitivity, ROC, IDI, and NRI analyses to assess the consistency and within-sample discriminatory performance of the biomarker combinations.

Several limitations should be acknowledged. First, the single-center cross-sectional design precluded assessment of temporal or causal relationships. Second, recruitment from hepatology clinics and health examination settings may have introduced selection bias and limited generalizability. Third, residual confounding may remain despite multivariable adjustment, particularly for lifestyle factors, medication use, genetic background, and socioeconomic status. Some laboratory covariates may also lie within the biological pathway linking liver disease and Lp(a), raising the possibility of overadjustment. Fourth, CAP and LSM were used instead of liver histology, and LSM-defined stiffness elevation is not equivalent to biopsy-confirmed fibrosis. Finally, although the lipid–inflammation combined models showed better discriminatory performance than individual markers and established noninvasive fibrosis scores, their clinical applicability requires further validation in external cohorts. Therefore, these models may serve as supportive tools for cross-sectional assessment but should not replace established imaging methods or histological evaluation.

## 5 Conclusion

Overall, among Chinese adults, higher Lp(a) levels were associated with lower odds of prevalent NAFLD and prevalent NAFLD with elevated liver stiffness, as well as lower CAP and LSM values. Combining Lp(a) with blood cell-derived inflammatory indices may provide complementary information for distinguishing individuals with and without prevalent hepatic steatosis and elevated liver stiffness. These findings suggest that lipid–inflammatory marker combinations may serve as supplementary markers for the noninvasive cross-sectional assessment of prevalent NAFLD and elevated liver stiffness.

## Data Availability

The raw data supporting the conclusions of this article will be made available by the authors, without undue reservation.

## Glossary

Lp(a): lipoprotein(a)
NAFLD: non-alcoholic fatty liver disease
BMI: body mass index
CAP: controlled attenuation parameter
LSM: liver stiffness measurement
ROC: receiver operating characteristic
AUC: area under the receiver operating characteristic curve
OR: odds ratio
CI: confidence interval
PPV: positive predictive value
NPV: negative predictive value
SIRI: systemic inflammation response index
SII: systemic immune-inflammation index
AISI: aggregate index of systemic inflammation
hs-CRP: high-sensitivity C-reactive protein
DM: diabetes mellitus
SBP: systolic blood pressure
DBP: diastolic blood pressure
WBC: white blood cell
NEU: neutrophil count
LYM: lymphocyte count
MONO: monocyte count
PLT: platelet count
TP: total protein
ALB: albumin
GLB: globulin
AGR: albumin/globulin ratio
ALT: alanine aminotransferase
AST: aspartate aminotransferase
GGT: gamma-glutamyl transferase
TBIL: total bilirubin
DBIL: direct bilirubin
IBIL: indirect bilirubin
TG: triglycerides
TC: total cholesterol
HDL-C: high-density lipoprotein cholesterol
LDL-C: low-density lipoprotein cholesterol
FPG: fasting plasma glucose
UA: uric acid
eGFR: estimated glomerular filtration rate
RCS: restricted cubic spline
VIF: variance inflation factor
IDI: integrated discrimination improvement
NRI: net reclassification improvement
FIB-4: fibrosis-4 index
APRI: aspartate aminotransferase-to-platelet ratio index
NFS: NAFLD fibrosis score
RERI: relative excess risk due to interaction
AP: attributable proportion due to interaction
SI: synergy index
and NASH: nonalcoholic steatohepatitis.
