# Supplementary material for: Joint associations of lipoprotein(a) and systemic inflammatory indices with FibroTouch-derived hepatic steatosis and liver stiffness in Chinese adults
Source: Front Immunol. 2026 Aug 21;17:1926199. doi: 10.3389/fimmu.2026.1926199 (PMC13541750; doi:10.3389/fimmu.2026.1926199)
Supplement: Supplementary file 1 [file SupplementaryFile1.docx]

**Supplementary Materials**

**Figure S1. English version of the basic case information form used for participant data collection.**

**Figure S2. Distribution of Lp(a) concentrations according to NAFLD and elevated liver stiffness status.**

**Supplementary Tables**

**Table S1. Baseline characteristics of participants according to NAFLD status.**

| **Variables** | **Total (n=1345)** | **Non-NAFLD (n=477)** | **NAFLD (n=868)** | ***P*** |
| --- | --- | --- | --- | --- |
| Age (years) | 43.00 (29.00, 53.00) | 41.00 (28.00, 50.00) | 44.00 (30.75, 54.00) | <0.001 |
| **Sex, n (%)** |  |  |  | 0.686 |
| Male | 698 (51.90) | 244 (51.15) | 454 (52.30) |  |
| Female | 647 (48.10) | 233 (48.85) | 414 (47.70) |  |
| BMI (kg/m²) | 25.67 ± 2.69 | 25.15 ± 2.63 | 25.96 ± 2.69 | <0.001 |
| SBP (mmHg) | 136.91 ± 12.04 | 136.00 ± 12.02 | 137.41 ± 12.03 | 0.041 |
| DBP (mmHg) | 84.97 ± 8.72 | 84.93 ± 8.57 | 84.99 ± 8.81 | 0.913 |
| **Hypertension, n (%)** |  |  |  | 0.031 |
| No | 562 (41.78) | 218 (45.70) | 344 (39.63) |  |
| Yes | 783 (58.22) | 259 (54.30) | 524 (60.37) |  |
| **DM, n (%)** |  |  |  | 0.770 |
| 0 | 636 (47.29) | 223 (46.75) | 413 (47.58) |  |
| 1 | 709 (52.71) | 254 (53.25) | 455 (52.42) |  |
| WBC (×10⁹/L) | 6.82 (5.65, 8.37) | 6.52 (5.40, 7.91) | 7.01 (5.73, 8.62) | <0.001 |
| NEU (×10⁹/L) | 4.33 (3.35, 5.92) | 3.96 (3.21, 5.10) | 4.58 (3.47, 6.32) | <0.001 |
| LYM (×10⁹/L) | 1.79 (1.41, 2.36) | 1.91 (1.56, 2.52) | 1.72 (1.33, 2.28) | <0.001 |
| MONO (×10⁹/L) | 0.34 (0.25, 0.49) | 0.36 (0.25, 0.48) | 0.33 (0.24, 0.50) | 0.373 |
| PLT (×10⁹/L) | 198.00 (150.35, 255.80) | 193.00 (141.00, 248.00) | 199.70 (154.94, 261.19) | 0.012 |
| SIRI | 0.83 (0.54, 1.29) | 0.74 (0.50, 1.09) | 0.90 (0.58, 1.41) | <0.001 |
| SII | 450.33 (327.65, 673.85) | 399.03 (313.09, 531.64) | 507.19 (338.41, 827.00) | <0.001 |
| AISI | 162.86 (100.52, 267.62) | 141.82 (94.33, 221.00) | 176.43 (102.57, 317.19) | <0.001 |
| hs-CRP (mg/L) | 4.32 (1.42, 15.01) | 3.42 (1.33, 13.76) | 4.76 (1.50, 15.18) | 0.281 |
| CAP (dB/m) | 260.26 ± 35.57 | 219.71 ± 16.26 | 282.54 ± 20.37 | <0.001 |
| LSM (kPa) | 7.84 ± 2.79 | 7.59 ± 2.68 | 7.98 ± 2.84 | 0.014 |
| TP (g/L) | 68.19 ± 9.32 | 68.17 ± 9.14 | 68.21 ± 9.43 | 0.945 |
| ALB (g/L) | 41.69 ± 7.11 | 41.70 ± 6.83 | 41.68 ± 7.26 | 0.964 |
| GLB (g/L) | 26.05 ± 4.74 | 25.99 ± 4.75 | 26.08 ± 4.74 | 0.729 |
| AGR | 1.63 ± 0.34 | 1.64 ± 0.34 | 1.63 ± 0.34 | 0.472 |
| ALT (U/L) | 57.30 (45.01, 69.00) | 56.30 (41.78, 68.90) | 58.66 (47.49, 69.09) | 0.006 |
| AST (U/L) | 43.11 (34.00, 52.92) | 41.00 (32.00, 51.99) | 46.09 (37.23, 54.30) | <0.001 |
| ALT/AST | 1.31 (1.05, 1.60) | 1.30 (1.05, 1.60) | 1.33 (1.03, 1.60) | 0.906 |
| GGT (U/L) | 39.00 (21.00, 81.73) | 41.00 (21.00, 91.00) | 37.00 (21.00, 75.00) | 0.104 |
| TBIL (μmol/L) | 20.00 (13.74, 31.24) | 19.42 (13.88, 30.66) | 20.30 (13.71, 31.76) | 0.634 |
| DBIL (μmol/L) | 5.20 (3.40, 9.80) | 5.17 (3.40, 9.32) | 5.20 (3.35, 10.35) | 0.627 |
| IBIL (μmol/L) | 13.04 (8.90, 19.70) | 13.00 (9.32, 20.15) | 13.10 (8.80, 19.30) | 0.469 |
| TG (mmol/L) | 1.82 (0.98, 2.42) | 1.63 (0.92, 2.30) | 1.92 (1.03, 2.45) | 0.001 |
| TC (mmol/L) | 5.08 (3.78, 6.20) | 4.77 (3.56, 5.90) | 5.24 (3.97, 6.41) | <0.001 |
| HDL (mmol/L) | 1.39 (1.05, 1.81) | 1.53 (1.12, 1.97) | 1.33 (1.04, 1.72) | <0.001 |
| LDL (mmol/L) | 3.23 (2.46, 4.05) | 3.20 (2.42, 4.12) | 3.24 (2.46, 4.02) | 0.801 |
| Lp(a) (mg/L) | 276.87 (192.08, 367.29) | 329.88 (236.28, 409.51) | 254.94 (179.34, 333.89) | <0.001 |
| FPG (mmol/L) | 6.22 (5.04, 8.23) | 6.21 (5.06, 8.33) | 6.25 (5.01, 8.14) | 0.976 |
| UA (μmol/L) | 362.86 ± 96.90 | 364.86 ± 95.02 | 361.76 ± 97.95 | 0.575 |
| eGFR (mL/min/1.73 m²) | 85.45 ± 19.19 | 87.61 ± 15.85 | 84.26 ± 20.71 | <0.001 |

**Notes:** Data are presented as mean ± standard deviation, median (interquartile range), or number (percentage), as appropriate. Participants were grouped according to NAFLD status. Continuous variables presented as means were compared using the independent-samples t test (Welch correction when variances were unequal); continuous variables presented as medians were compared using the Mann-Whitney U test. Categorical variables were compared using the chi-square test or Fisher's exact test, as appropriate. Analyses were based on available data.

**Abbreviations:** NAFLD, nonalcoholic fatty liver disease; BMI, body mass index; SBP, systolic blood pressure; DBP, diastolic blood pressure; DM, diabetes mellitus; WBC, white blood cell count; NEU, neutrophil count; LYM, lymphocyte count; MONO, monocyte count; PLT, platelet count; SIRI, systemic inflammation response index; SII, systemic immune-inflammation index; AISI, aggregate index of systemic inflammation; hs-CRP, high-sensitivity C-reactive protein; CAP, controlled attenuation parameter; LSM, liver stiffness measurement; TP, total protein; ALB, albumin; GLB, globulin; AGR, albumin/globulin ratio; ALT, alanine aminotransferase; AST, aspartate aminotransferase; GGT, gamma-glutamyl transferase; TBIL, total bilirubin; DBIL, direct bilirubin; IBIL, indirect bilirubin; TG, triglycerides; TC, total cholesterol; HDL, high-density lipoprotein cholesterol; LDL, low-density lipoprotein cholesterol; Lp(a), lipoprotein(a); FPG, fasting plasma glucose; UA, uric acid; eGFR, estimated glomerular filtration rate.

**Table S2. Baseline characteristics of participants with NAFLD according to elevated liver stiffness status.**

| Variables | Total (n = 868) | NAFLD without elevated liver stiffness (n = 235) | NAFLD with elevated liver stiffness (n = 633) | *P* |
| --- | --- | --- | --- | --- |
| Age (years) | 44.00 (30.75, 54.00) | 39.00 (24.00, 48.00) | 46.00 (33.00, 56.00) | <0.001 |
| Sex, n (%) |  |  |  | 0.216 |
| Male | 454 (52.30) | 131 (55.74) | 323 (51.03) |  |
| Female | 414 (47.70) | 104 (44.26) | 310 (48.97) |  |
| BMI (kg/m²) | 25.96 ± 2.69 | 25.74 ± 2.62 | 26.04 ± 2.71 | 0.145 |
| SBP (mmHg) | 137.41 ± 12.03 | 136.71 ± 11.79 | 137.67 ± 12.12 | 0.299 |
| DBP (mmHg) | 84.99 ± 8.81 | 84.37 ± 8.88 | 85.22 ± 8.77 | 0.205 |
| Hypertension, n (%) |  |  |  | 0.166 |
| No | 344 (39.63) | 102 (43.40) | 242 (38.23) |  |
| Yes | 524 (60.37) | 133 (56.60) | 391 (61.77) |  |
| DM, n (%) |  |  |  | 0.272 |
| No | 413 (47.58) | 119 (50.64) | 294 (46.45) |  |
| Yes | 455 (52.42) | 116 (49.36) | 339 (53.55) |  |
| WBC (×10⁹/L) | 7.01 (5.73, 8.62) | 6.94 (5.57, 8.62) | 7.07 (5.75, 8.62) | 0.519 |
| NEU (×10⁹/L) | 4.58 (3.47, 6.32) | 4.39 (3.34, 6.25) | 4.61 (3.52, 6.38) | 0.117 |
| LYM (×10⁹/L) | 1.72 (1.33, 2.28) | 1.83 (1.35, 2.49) | 1.69 (1.32, 2.19) | 0.022 |
| MONO (×10⁹/L) | 0.33 (0.24, 0.50) | 0.32 (0.24, 0.45) | 0.34 (0.24, 0.52) | 0.039 |
| PLT (×10⁹/L) | 199.70 (154.94, 261.19) | 188.50 (147.50, 252.25) | 204.10 (157.00, 265.00) | 0.073 |
| SIRI | 0.90 (0.58, 1.41) | 0.79 (0.55, 1.13) | 0.94 (0.59, 1.48) | <0.001 |
| SII | 507.19 (338.41, 827.00) | 437.13 (303.47, 763.27) | 536.48 (357.50, 858.52) | <0.001 |
| AISI | 176.43 (102.57, 317.19) | 147.28 (95.94, 249.90) | 191.19 (106.77, 351.04) | <0.001 |
| hs-CRP (mg/L) | 4.76 (1.50, 15.18) | 3.47 (1.04, 9.45) | 5.50 (1.73, 17.06) | 0.010 |
| CAP (dB/m) | 282.54 ± 20.37 | 284.65 ± 20.45 | 281.76 ± 20.29 | 0.064 |
| LSM (kPa) | 9.19 ± 2.54 | 5.92 ± 1.42 | 10.41 ± 1.63 | <0.001 |
| TP (g/L) | 68.21 ± 9.43 | 68.52 ± 9.35 | 68.09 ± 9.46 | 0.551 |
| ALB (g/L) | 41.68 ± 7.26 | 41.75 ± 7.15 | 41.65 ± 7.31 | 0.866 |
| GLB (g/L) | 26.08 ± 4.74 | 26.55 ± 4.51 | 25.92 ± 4.82 | 0.086 |
| AGR | 1.63 ± 0.34 | 1.61 ± 0.33 | 1.64 ± 0.34 | 0.364 |
| ALT (U/L) | 56.30 (41.78, 68.90) | 56.63 (41.45, 69.06) | 56.15 (42.05, 68.83) | 0.941 |
| AST (U/L) | 41.00 (32.00, 51.99) | 38.58 (30.98, 50.20) | 42.00 (32.27, 52.09) | 0.037 |
| ALT/AST | 1.30 (1.05, 1.60) | 1.32 (1.08, 1.66) | 1.29 (1.02, 1.60) | 0.252 |
| GGT (U/L) | 37.00 (21.00, 75.00) | 37.00 (21.00, 74.00) | 37.00 (21.00, 75.00) | 0.988 |
| TBIL (μmol/L) | 20.30 (13.71, 31.76) | 19.41 (13.20, 31.39) | 20.49 (13.80, 32.10) | 0.651 |
| DBIL (μmol/L) | 5.20 (3.35, 10.35) | 4.98 (3.30, 9.72) | 5.30 (3.40, 10.54) | 0.717 |
| IBIL (μmol/L) | 13.10 (8.80, 19.30) | 12.30 (8.85, 18.98) | 13.25 (8.83, 19.70) | 0.485 |
| TG (mmol/L) | 1.92 (1.03, 2.45) | 1.57 (0.90, 2.23) | 2.04 (1.15, 2.55) | <0.001 |
| TC (mmol/L) | 5.24 (3.97, 6.41) | 4.80 (3.63, 5.49) | 5.46 (4.07, 6.58) | <0.001 |
| HDL (mmol/L) | 1.33 (1.04, 1.72) | 1.31 (1.04, 1.71) | 1.33 (1.03, 1.73) | 0.790 |
| LDL (mmol/L) | 3.24 (2.46, 4.02) | 3.08 (2.38, 3.94) | 3.26 (2.47, 4.03) | 0.295 |
| Lp(a) (mg/L) | 254.94 (179.34, 333.89) | 279.45 (211.47, 364.11) | 249.00 (166.64, 319.59) | <0.001 |
| FPG (mmol/L) | 6.25 (5.01, 8.14) | 6.05 (5.11, 7.83) | 6.34 (4.98, 8.38) | 0.048 |
| UA (μmol/L) | 361.76 ± 97.95 | 365.39 ± 102.20 | 360.41 ± 96.38 | 0.506 |
| eGFR (mL/min/1.73 m²) | 84.26 ± 20.71 | 88.12 ± 16.16 | 82.83 ± 22.00 | <0.001 |

**Notes:** Data are presented as mean ± standard deviation, median (interquartile range), or number (percentage), as appropriate. Continuous variables shown as mean ± standard deviation were compared using the independent-samples t test (Welch correction for unequal variances); variables shown as medians were compared using the Mann–Whitney U test. Categorical variables were compared using the chi-square test or Fisher’s exact test, as appropriate. Analyses were based on available data.

**Table S3. Mediation analysis of potential mediators in the association between Lp(a) and prevalent NAFLD**

| Mediation pathway | Total effect (c) | Path a | Path b | Indirect effect (a x b) | Bootstrap SE | z value | P value | 95% bootstrap CI | Direct effect (c') | Conclusion |
| --- | --- | --- | --- | --- | --- | --- | --- | --- | --- | --- |
| Lp(a) -> ALT -> NAFLD | -0.4480 | 0.7913 | -0.0036 | -0.0029 | 0.0034 | -0.846 | 0.398 | -0.0110 to 0.0025 | -0.4458 | No significant mediation |
| Lp(a) -> AST -> NAFLD | -0.4480 | 0.6363 | -0.0118 | -0.0075 | 0.0052 | -1.435 | 0.151 | -0.0193 to 0.0011 | -0.4427 | No significant mediation |
| Lp(a) -> TC -> NAFLD | -0.4480 | -0.1055 | 0.1156 | -0.0122 | 0.0059 | -2.078 | 0.380 | -0.0251 to -0.0025 | -0.4393 | No significant mediation |
| Lp(a) -> TG -> NAFLD | -0.4480 | -0.0553 | 0.0903 | -0.0050 | 0.0043 | -1.153 | 0.249 | -0.0152 to 0.0019 | -0.4434 | No significant mediation |
| Lp(a) -> HDL-C -> NAFLD | -0.4480 | 0.0057 | -0.6850 | -0.0039 | 0.0075 | -0.525 | 0.600 | -0.0193 to 0.0106 | -0.4535 | No significant mediation |
| Lp(a) -> LDL-C -> NAFLD | -0.4480 | 0.0082 | 0.0129 | 0.0001 | 0.0015 | 0.070 | 0.944 | -0.0029 to 0.0038 | -0.4482 | No significant mediation |
| Lp(a) -> UA -> NAFLD | -0.4480 | -0.4277 | -0.0004 | 0.0002 | 0.0016 | 0.102 | 0.919 | -0.0032 to 0.0037 | -0.4482 | No significant mediation |
| Lp(a) -> eGFR -> NAFLD | -0.4480 | 0.0086 | -0.0090 | -0.0001 | 0.0040 | -0.019 | 0.985 | -0.0082 to 0.0082 | -0.4511 | No significant mediation |
| Lp(a) -> GGT -> NAFLD | -0.4435 | 6.3832 | -0.0006 | -0.0040 | 0.0052 | -0.765 | 0.444 | -0.0147 to 0.0069 | -0.4396 | No significant mediation |
| Lp(a) -> ALB -> NAFLD | -0.4504 | 0.1721 | 0.0014 | 0.0002 | 0.0020 | 0.121 | 0.904 | -0.0036 to 0.0049 | -0.4507 | No significant mediation |
| Lp(a) -> GLB -> NAFLD | -0.4454 | 0.2230 | 0.0125 | 0.0028 | 0.0036 | 0.776 | 0.438 | -0.0027 to 0.0115 | -0.4489 | No significant mediation |
| Lp(a) -> DBIL -> NAFLD | -0.4365 | 1.2011 | 0.0010 | 0.0012 | 0.0027 | 0.450 | 0.653 | -0.0034 to 0.0082 | -0.4380 | No significant mediation |
| Lp(a) -> IBIL -> NAFLD | -0.4414 | 0.0278 | 0.0005 | 0.0000 | 0.0016 | 0.008 | 0.993 | -0.0034 to 0.0034 | -0.4415 | No significant mediation |

Notes: Each mediator was evaluated in a separate single-mediator model using complete cases. Lp(a) was analyzed per 100 mg/L increase. All models were adjusted for age and gender. Path a was estimated using linear regression; total effect (c), path b, and direct effect (c') were estimated using binary logistic regression and are reported as log-odds coefficients. The indirect effect was calculated as a x b on the logit scale. Bootstrap SE and percentile 95% CI were obtained from 5,000 nonparametric bootstrap resamples. z and P values were calculated from the indirect effect divided by its bootstrap SE; mediation was considered significant when the bootstrap 95% CI excluded 0.

**Table S4. Pearson correlations between laboratory parameters and composite inflammatory indices.**

| **Laboratory parameter** | **SIRI, r** | **SII, r** | **AISI, r** | **Maximum \|r\|** | **Substantial pairwise collinearity** |
| --- | --- | --- | --- | --- | --- |
| ALT | -0.012 | 0.032 | 0.026 | 0.032 | No |
| AST | -0.029 | -0.018 | -0.011 | 0.029 | No |
| TC | 0.110^**^ | 0.113^**^ | 0.115^**^ | 0.115 | No |
| TG | 0.099^**^ | 0.070^*^ | 0.087^**^ | 0.099 | No |
| HDL-C | -0.042 | -0.102^**^ | -0.065^*^ | 0.102 | No |
| LDL-C | 0.010 | 0.009 | 0.005 | 0.010 | No |
| ALB | -0.075^**^ | -0.003 | -0.032 | 0.075 | No |
| GLB | -0.036 | 0.003 | -0.021 | 0.036 | No |

**Notes:** Values are Pearson correlation coefficients. * P < 0.05; ** P < 0.01. Substantial pairwise collinearity was defined as |r| ≥ 0.70. No correlation reached this threshold; the largest absolute coefficient was 0.115. Pairwise correlations do not replace variance inflation factor assessment in multivariable models.

**Table S5. Collinearity diagnostics of laboratory parameters across composite inflammatory index models.**

| **Laboratory parameter** | **SIRI model VIF (Tolerance)** | **SII model VIF (Tolerance)** | **AISI model VIF (Tolerance)** |
| --- | --- | --- | --- |
| ALT | 1.530 (0.654) | 1.534 (0.652) | 1.533 (0.652) |
| AST | 1.532 (0.653) | 1.534 (0.652) | 1.533 (0.652) |
| TC | 1.242 (0.805) | 1.245 (0.803) | 1.244 (0.804) |
| TG | 1.237 (0.808) | 1.234 (0.810) | 1.235 (0.809) |
| HDL-C | 1.012 (0.988) | 1.020 (0.980) | 1.014 (0.986) |
| LDL-C | 1.002 (0.998) | 1.002 (0.998) | 1.002 (0.998) |
| ALB | 1.088 (0.919) | 1.083 (0.924) | 1.084 (0.923) |
| GLB | 1.088 (0.919) | 1.088 (0.919) | 1.088 (0.919) |

**Notes:** Values are presented as VIF (Tolerance). Only collinearity diagnostics involving laboratory parameters and each composite inflammatory index model are shown; collinearity among SIRI, SII, and AISI was not evaluated in this table. All VIF values were <5 and all tolerance values were >0.10, indicating no evidence of substantial multicollinearity.

**Table S6. Discriminatory performance and optimal cutoff values of comparator indices added to the fully adjusted base model for prevalent NAFLD.**

| **Comparator index** | **AUC (95% CI)** | **Standard error** | ***p*** | **Youden index** | **Sensitivity** | **Specificity** | **Cutoff** |
| --- | --- | --- | --- | --- | --- | --- | --- |
| **NAFLD** |  |  |  |  |  |  |  |
| Base Model 3 | 0.586 (0.554-0.617) | 0.014 | <0.001 | 0.292 | 0.753 | 0.539 | 0.672 |
| + Lp(a) | 0.707 (0.676–0.737) | 0.015 | <0.001 | 0.322 | 0.586 | 0.736 | 0.670 |
| + SIRI | 0.676 (0.645–0.706) | 0.016 | <0.001 | 0.275 | 0.480 | 0.795 | 0.687 |
| + SII | 0.727 (0.699–0.755) | 0.014 | <0.001 | 0.376 | 0.524 | 0.852 | 0.698 |
| + AISI | 0.691 (0.661–0.721) | 0.015 | <0.001 | 0.323 | 0.576 | 0.748 | 0.654 |
| + Lp(a) + SIRI | 0.717 (0.687–0.746) | 0.015 | <0.001 | 0.345 | 0.659 | 0.686 | 0.635 |
| + Lp(a) + SII | 0.762 (0.735–0.789) | 0.014 | <0.001 | 0.422 | 0.619 | 0.802 | 0.671 |
| + Lp(a) + AISI | 0.727 (0.698–0.756) | 0.015 | <0.001 | 0.357 | 0.566 | 0.790 | 0.681 |
| + FIB-4 | 0.664 (0.633–0.695) | 0.016 | <0.001 | 0.271 | 0.540 | 0.731 | 0.669 |
| + APRI | 0.654 (0.623–0.686) | 0.016 | <0.001 | 0.232 | 0.594 | 0.638 | 0.643 |
| + NFS | 0.665 (0.634–0.696) | 0.016 | <0.001 | 0.285 | 0.489 | 0.795 | 0.682 |

**Note:** The optimal cutoff was determined by maximizing the Youden index (sensitivity + specificity − 1). All values are presented as decimals except p values.

**Table S7. Discriminatory performance and optimal cutoff values of comparator indices added to the fully adjusted base model for prevalent NAFLD with elevated liver stiffness.**

| **Comparator index** | **AUC (95% CI)** | **Standard error** | ***p*** | **Youden index** | **Sensitivity** | **Specificity** | **Optimal cutoff** |
| --- | --- | --- | --- | --- | --- | --- | --- |
| **NAFLD with elevated liver stiffness** |  |  |  |  |  |  |  |
| Base Model 3 | 0.632 (0.595-0.669) | 0.016 | <0.001 | 0.409 | 0.845 | 0.564 | 0.579 |
| + Lp(a) | 0.790 (0.759–0.821) | 0.016 | <0.001 | 0.443 | 0.614 | 0.830 | 0.571 |
| + SIRI | 0.785 (0.753–0.817) | 0.016 | <0.001 | 0.435 | 0.504 | 0.931 | 0.672 |
| + SII | 0.768 (0.735–0.801) | 0.017 | <0.001 | 0.429 | 0.564 | 0.865 | 0.599 |
| + AISI | 0.782 (0.749–0.814) | 0.016 | <0.001 | 0.444 | 0.543 | 0.901 | 0.622 |
| + Lp(a) + SIRI | 0.811 (0.781–0.840) | 0.015 | <0.001 | 0.486 | 0.713 | 0.774 | 0.494 |
| + Lp(a) + SII | 0.799 (0.768–0.830) | 0.016 | <0.001 | 0.476 | 0.689 | 0.786 | 0.509 |
| + Lp(a) + AISI | 0.809 (0.779–0.839) | 0.015 | <0.001 | 0.491 | 0.687 | 0.804 | 0.531 |
| + FIB-4 | 0.760 (0.727–0.794) | 0.017 | <0.001 | 0.405 | 0.522 | 0.883 | 0.615 |
| + APRI | 0.751 (0.717–0.785) | 0.017 | <0.001 | 0.398 | 0.540 | 0.858 | 0.577 |
| + NFS | 0.762 (0.729–0.795) | 0.017 | <0.001 | 0.404 | 0.572 | 0.832 | 0.565 |

**Note:** The optimal cutoff was determined by maximizing the Youden index (sensitivity + specificity − 1).

**Abbreviations:** AISI, aggregate index of systemic inflammation; APRI, aspartate aminotransferase-to-platelet ratio index; AUC, area under the receiver operating characteristic curve; FIB-4, fibrosis-4 index; Lp(a), lipoprotein(a); NAFLD, nonalcoholic fatty liver disease; NFS, NAFLD fibrosis score; SII, systemic immune-inflammation index; SIRI, systemic inflammation response index.

**Table S8. IDI and NRI for comparator indices added to the fully adjusted base model for prevalent NAFLD.**

| **Comparator index** | **IDI (95% CI), %** | **NRI (95% CI), %** |
| --- | --- | --- |
| **NAFLD** |  |  |
| **Base model (Model 3)** | – | – |
| + Lp(a) | 4.60 (3.40–5.80) | 43.07 (31.56–54.58) |
| + SIRI | 1.50 (0.80–2.20) | 26.99 (15.94–38.03) |
| + SII | 7.40 (6.00–8.70) | 40.07 (28.91–51.23) |
| + AISI | 2.90 (2.00–3.80) | 26.42 (15.40–37.44) |
| + Lp(a) + SIRI | 5.90 (4.60–7.30) | 40.28 (28.74–51.83) |
| + Lp(a) + SII | 11.80 (10.00–13.50) | 59.56 (48.51–70.61) |
| + Lp(a) + AISI | 7.10 (5.70–8.60) | 45.07 (33.67–56.47) |
| + FIB-4 | 4.00 (1.60–6.40) | 12.87 (4.20–21.54) |
| + APRI | 3.70 (1.50–5.90) | 10.88 (2.22–19.53) |
| + NFS | 3.80 (1.60–6.00) | 12.37 (3.66–21.08) |

**Note:** The base model (Model 3) was adjusted for sex, diabetes mellitus, hypertension, age, BMI, ALT, AST, TC, TG, HDL-C, LDL-C, UA, eGFR, GGT, ALB, GLB, DBIL, and IBIL. IDI estimates were converted from proportions to percentages.

**Table S9. IDI and NRI for comparator indices added to the fully adjusted base model for prevalent NAFLD with elevated liver stiffness.**

| **Comparator index** | **IDI (95% CI), %** | **NRI (95% CI), %** |
| --- | --- | --- |
| **NAFLD with elevated liver stiffness** |  |  |
| **Base model (Model 3)** | – | – |
| + Lp(a) | 4.80 (3.30–6.40) | 47.54 (33.89–61.20) |
| + SIRI | 3.60 (2.30–4.90) | 35.42 (22.04–48.79) |
| + SII | 1.20 (0.50–2.00) | 23.19 (9.50–36.88) |
| + AISI | 3.40 (2.20–4.70) | 33.26 (20.01–46.52) |
| + Lp(a)–SIRI | 8.20 (6.30–10.10) | 56.16 (42.65–69.66) |
| + Lp(a)–SII | 6.20 (4.50–7.90) | 45.41 (31.71–59.12) |
| + Lp(a)–AISI | 8.10 (6.20–10.00) | 51.47 (37.88–65.06) |
| + FIB-4 | 0.30 (−0.10–0.70) | 18.56 (4.85–32.28) |
| + APRI | 0.20 (−0.10–0.50) | 8.85 (−4.81–22.52) |
| + NFS | 0.60 (0.00–1.10) | 10.03 (−3.94–24.00) |

**Note:** The base model (Model 3) was adjusted for sex, diabetes mellitus, hypertension, age, BMI, ALT, AST, TC, TG, HDL-C, LDL-C, UA, eGFR, GGT, ALB, GLB, DBIL, and IBIL. IDI estimates were converted from proportions to percentages.

| Model | NAFLD (CAP ≥275 dB/m) | | | | | NAFLD with elevated liver stiffness (CAP ≥275 dB/m and LSM ≥8 kPa) | | | | |
| --- | --- | --- | --- | --- | --- | --- | --- | --- | --- | --- |
|  | n | Events, n | Event rate, % | OR (95% CI) | P-value | n | Events, n | Event rate, % | OR (95% CI) | *P-value* |
| Model 1 | 1345 | 544 | 40.4 | 0.36 (0.28-0.45) | <0.001 | 1345 | 275 | 20.4 | 0.30 (0.22-0.41) | <0.001 |
| Model 2 | 1345 | 544 | 40.4 | 0.38 (0.30-0.47) | <0.001 | 1345 | 275 | 20.4 | 0.31 (0.23-0.43) | <0.001 |
| Model 3 | 1205 | 487 | 40.4 | 0.37 (0.28-0.47) | <0.001 | 1205 | 246 | 20.4 | 0.29 (0.20-0.42) | <0.001 |

**Supplementary Table S10. Sensitivity analysis of the association between Lp(a) ≥300 mg/L and prevalent NAFLD-related outcomes using a CAP cutoff of 275 dB/m.**

Note: Elevated Lp(a) was defined as Lp(a) ≥300 mg/L, with Lp(a) <300 mg/L as the reference. NAFLD was defined as CAP ≥275 dB/m, and NAFLD with elevated liver stiffness was defined as CAP ≥275 dB/m together with LSM ≥8 kPa. Model 1 was unadjusted. Model 2 was adjusted for age, sex, hypertension, and BMI. Model 3 was further adjusted for diabetes, ALT, AST, TC, TG, HDL-C, LDL-C, UA, eGFR, GGT, ALB, GLB, DBIL, and IBIL. The sample size in Model 3 was reduced to 1,205 because the fully adjusted analysis was restricted to participants with complete data for all included covariates. OR, odds ratio; CI, confidence interval; NAFLD, nonalcoholic fatty liver disease; CAP, controlled attenuation parameter; LSM, liver stiffness measurement; Lp(a), lipoprotein(a).

**Supplementary Table S11. Sensitivity analysis of the association between Lp(a) ≥300 mg/L and prevalent NAFLD-related outcomes using a CAP cutoff of 288 dB/m.**

| Model | NAFLD (CAP ≥288 dB/m) | | | | | NAFLD with elevated liver stiffness (CAP ≥288 dB/m and LSM ≥8 kPa) | | | | |
| --- | --- | --- | --- | --- | --- | --- | --- | --- | --- | --- |
|  | n | Events, n | Event rate, % | OR (95% CI) | *P-value* | n | Events, n | Event rate, % | OR (95% CI) | *P-value* |
| Model 1 | 1345 | 319 | 23.7 | 0.29 (0.22-0.39) | *<0.001* | 1345 | 149 | 11.1 | 0.39 (0.27-0.58) | <0.001 |
| Model 2 | 1345 | 319 | 23.7 | 0.31 (0.23-0.41) | *<0.001* | 1345 | 149 | 11.1 | 0.44 (0.29-0.65) | <0.001 |
| Model 3 | 1205 | 287 | 23.8 | 0.31 (0.22-0.42) | *<0.001* | 1205 | 133 | 11.0 | 0.51 (0.33-0.78) | 0.002 |

Note: Elevated Lp(a) was defined as Lp(a) ≥300 mg/L, with Lp(a) <300 mg/L as the reference. NAFLD was defined as CAP ≥288 dB/m, and NAFLD with elevated liver stiffness was defined as CAP ≥288 dB/m together with LSM ≥8 kPa. Model 1 was unadjusted. Model 2 was adjusted for age, sex, hypertension, and BMI. Model 3 was further adjusted for diabetes, ALT, AST, TC, TG, HDL-C, LDL-C, UA, eGFR, GGT, ALB, GLB, DBIL, and IBIL. The sample size in Model 3 was reduced to 1,205 because the fully adjusted analysis was restricted to participants with complete data for all included covariates. OR, odds ratio; CI, confidence interval; NAFLD, nonalcoholic fatty liver disease; CAP, controlled attenuation parameter; LSM, liver stiffness measurement; Lp(a), lipoprotein(a).

**Table S12. Cross-sectional associations of Lp(a) with prevalent NAFLD and prevalent NAFLD with elevated liver stiffness after exclusion of extreme Lp(a) values.**

| **Variables** | **Model 1** | | **Model 2** | | **Model 3** | |
| --- | --- | --- | --- | --- | --- | --- |
|  | **OR (95% CI)** | ***P-value*** | **OR (95% CI)** | ***P-value*** | **OR (95% CI)** | ***P-value*** |
| **NAFLD** |  |  |  |  |  |  |
| Continuous Lp(a) | 0.64 (0.58–0.71) | **<0.001** | 0.65 (0.59–0.73) | **<0.001** | 0.67 (0.60–0.75) | **<0.001** |
| Lp(a) quartile |  |  |  |  |  |  |
| Q1 | Ref. |  | Ref. |  | Ref. |  |
| Q2 | 0.76 (0.54–1.08) | 0.128 | 0.79 (0.56–1.13) | 0.194 | 0.73 (0.50–1.07) | 0.111 |
| Q3 | 0.55 (0.39–0.77) | **<0.001** | 0.57 (0.41–0.81) | **0.001** | 0.55 (0.38–0.79) | **0.001** |
| Q4 | 0.27 (0.19–0.37) | **<0.001** | 0.29 (0.21–0.40) | **<0.001** | 0.30 (0.21–0.43) | **<0.001** |
| **NAFLD with elevated liver stiffness** |  |  |  |  |  |  |
| Continuous Lp(a) | 0.69 (0.63–0.76) | **<0.001** | 0.71 (0.64–0.78) | **<0.001** | 0.71 (0.63–0.79) | **<0.001** |
| Lp(a) quartile |  |  |  |  |  |  |
| Q1 | Ref. |  | Ref. |  | Ref. |  |
| Q2 | 0.65 (0.48–0.88) | **0.006** | 0.67 (0.49–0.93) | **0.015** | 0.71 (0.50–1.02) | 0.064 |
| Q3 | 0.59 (0.43–0.80) | **<0.001** | 0.61 (0.44–0.83) | **0.002** | 0.67 (0.47–0.95) | **0.026** |
| Q4 | 0.32 (0.23–0.44) | **<0.001** | 0.34 (0.25–0.47) | **<0.001** | 0.33 (0.23–0.48) | **<0.001** |

**Notes:** Logistic regression analyses were performed to evaluate the associations of Lp(a) with NAFLD and NAFLD with elevated liver stiffness. Lp(a) was analyzed both as a continuous variable and by quartiles, with Q1 used as the reference group. Data are presented as odds ratios with 95% confidence intervals. Model 1 was unadjusted. Model 2 was adjusted for sex, hypertension, age, and BMI. Model 3 was adjusted for sex, diabetes mellitus, hypertension, age, BMI, ALT, AST, TC, TG, HDL, LDL, UA, eGFR, GGT, ALB, GLB, DBIL, and IBIL. Abbreviations: OR, odds ratio; CI, confidence interval; NAFLD, nonalcoholic fatty liver disease; Lp(a), lipoprotein(a).

**Table S13. Joint associations of Lp(a) and systemic inflammatory index categories with prevalent NAFLD and prevalent NAFLD with elevated liver stiffness after exclusion of extreme Lp(a) values.**

| **Outcome** | **Joint exposure group** | **n** | **Events** | **Adjusted OR (95% CI)** | **P-value** |
| --- | --- | --- | --- | --- | --- |
| NAFLD | High Lp(a) + low SIRI | 317 | 162 | 1.00 (Reference) |  |
|  | Low Lp(a) + low SIRI | 280 | 187 | 1.87 (1.32–2.64) | <0.001 |
|  | High Lp(a) + high SIRI | 281 | 167 | 1.48 (1.06–2.08) | 0.023 |
|  | Low Lp(a) + high SIRI | 318 | 254 | 3.46 (2.40–5.00) | <0.001 |
|  | High Lp(a) + low SII | 302 | 142 | 1.00 (Reference) |  |
|  | Low Lp(a) + low SII | 296 | 199 | 2.07 (1.47–2.93) | <0.001 |
|  | High Lp(a) + high SII | 296 | 187 | 1.87 (1.33–2.63) | <0.001 |
|  | Low Lp(a) + high SII | 302 | 242 | 4.10 (2.81–5.98) | <0.001 |
|  | High Lp(a) + low AISI | 315 | 161 | 1.00 (Reference) |  |
|  | Low Lp(a) + low AISI | 283 | 195 | 1.97 (1.39–2.80) | <0.001 |
|  | High Lp(a) + high AISI | 283 | 168 | 1.38 (0.98–1.93) | 0.063 |
|  | Low Lp(a) + high AISI | 315 | 246 | 3.01 (2.10–4.32) | <0.001 |
| NAFLD with elevated liver stiffness | High Lp(a) + low SIRI | 317 | 104 | 1.00 (Reference) |  |
|  | Low Lp(a) + low SIRI | 280 | 134 | 1.91 (1.33–2.75) | <0.001 |
|  | High Lp(a) + high SIRI | 281 | 134 | 1.77 (1.23–2.55) | 0.002 |
|  | Low Lp(a) + high SIRI | 318 | 186 | 2.76 (1.93–3.95) | <0.001 |
|  | High Lp(a) + low SII | 302 | 112 | 1.00 (Reference) |  |
|  | Low Lp(a) + low SII | 296 | 142 | 1.47 (1.03–2.11) | 0.036 |
|  | High Lp(a) + high SII | 296 | 126 | 1.04 (0.72–1.49) | 0.835 |
|  | Low Lp(a) + high SII | 302 | 178 | 2.21 (1.54–3.18) | <0.001 |
|  | High Lp(a) + low AISI | 315 | 105 | 1.00 (Reference) |  |
|  | Low Lp(a) + low AISI | 283 | 138 | 1.97 (1.37–2.83) | <0.001 |
|  | High Lp(a) + high AISI | 283 | 133 | 1.66 (1.15–2.39) | 0.006 |
|  | Low Lp(a) + high AISI | 315 | 182 | 2.55 (1.78–3.65) | <0.001 |

**Notes:** Multivariable logistic regression analyses were performed to evaluate the associations of joint exposure to Lp(a) and systemic inflammatory indices with prevalent NAFLD and prevalent NAFLD with elevated liver stiffness. Lp(a), SIRI, SII, and AISI were dichotomized at their medians in the complete-case analytic sample (276.76, 0.81, 446.785, and 160.11, respectively). The high Lp(a) plus low inflammatory index group was used as the reference group. Models were adjusted for sex, diabetes mellitus, hypertension, age, BMI, ALT, AST, TC, TG, HDL-C, LDL-C, UA, eGFR, GGT, ALB, GLB, DBIL, and IBIL. Data are presented as adjusted odds ratios with 95% confidence intervals. The analysis included 1,196 participants after excluding Lp(a) values outside the central 99% interval and restricting the analysis to participants with complete covariate data.

**Table S14. Sensitivity analysis of the cross-sectional associations of Lp(a) ≥300 mg/L with prevalent NAFLD and prevalent NAFLD with elevated liver stiffness after exclusion of extreme Lp(a) values.**

| **Model** | **NAFLD** | | | | | **NAFLD with elevated liver stiffness** | | | | |
| --- | --- | --- | --- | --- | --- | --- | --- | --- | --- | --- |
|  | n | Events, n | Event rate, % | OR (95% CI) | *P-value* | n | Events, n | Event rate, % | OR (95% CI) | *P-value* |
| Model 1 | 1331 | 861 | 64.7 | 0.38 (0.30–0.48) | <0.001 | 1331 | 624 | 46.9 | 0.52 (0.42–0.65) | <0.001 |
| Model 2 | 1331 | 861 | 64.7 | 0.40 (0.32–0.50) | <0.001 | 1331 | 624 | 46.9 | 0.55 (0.43–0.69) | <0.001 |
| Model 3 | 1196 | 770 | 64.4 | 0.41 (0.32–0.53) | <0.001 | 1196 | 558 | 46.7 | 0.55 (0.43–0.72) | <0.001 |

**Note:** Analyses were conducted after excluding observations outside the central 99% interval of Lp(a), analyzed per 100 mg/L or according to the prespecified cutoff. Elevated Lp(a) was defined as Lp(a) ≥300 mg/L. Model 1 was unadjusted. Model 2 was adjusted for age, sex, hypertension, and BMI. Model 3 was further adjusted for diabetes, ALT, AST, TC, TG, HDL-C, LDL-C, UA, eGFR, GGT, ALB, GLB, DBIL, and IBIL. The sample size in Model 3 was reduced to 1,196 because the fully adjusted analysis was restricted to participants with complete data for all covariates included in the model. OR, odds ratio; CI, confidence interval; NAFLD, nonalcoholic fatty liver disease; Lp(a), lipoprotein(a).

**Table S15.** Sensitivity analysis of the associations of systemic inflammatory indices with prevalent NAFLD and NAFLD with elevated liver stiffness after exclusion of values outside the central 99% interval.

| **Inflammatory index** | **Outcome** | **Retained n** | **Model 1 OR (95% CI)** | **Model 2 OR (95% CI)** | **Model 3 OR (95% CI)** |
| --- | --- | --- | --- | --- | --- |
| SIRI | Prevalent NAFLD | 1331 | 1.61 (1.28–2.02) | 1.64 (1.31–2.07) | 1.65 (1.29–2.12) |
| SIRI | NAFLD with elevated liver stiffness | 1331 | 1.71 (1.38–2.13) | 1.75 (1.39–2.19) | 1.62 (1.26–2.09) |
| SII | Prevalent NAFLD | 1331 | 1.91 (1.52–2.40) | 1.95 (1.55–2.46) | 1.82 (1.42–2.35) |
| SII | NAFLD with elevated liver stiffness | 1331 | 1.35 (1.08–1.67) | 1.35 (1.08–1.69) | 1.23 (0.95–1.58) |
| AISI | Prevalent NAFLD | 1331 | 1.53 (1.22–1.91) | 1.49 (1.18–1.87) | 1.49 (1.16–1.91) |
| AISI | NAFLD with elevated liver stiffness | 1331 | 1.53 (1.23–1.90) | 1.47 (1.18–1.84) | 1.49 (1.16–1.92) |

**Notes:** Separate sensitivity datasets were created for SIRI, SII, and AISI by retaining observations within the index-specific central 99% interval (0.5th–99.5th percentiles) and excluding values in both tails. The median cutoffs established in the original complete-case analysis were retained: SIRI, 0.81; SII, 447.14; and AISI, 160.14. Odds ratios compare the high-index group (≥ cutoff) with the low-index group (< cutoff). Model 1 was unadjusted. Model 2 was adjusted for sex, hypertension, age, and BMI. Model 3 was further adjusted for diabetes mellitus, ALT, AST, TC, TG, HDL-C, LDL-C, UA, eGFR, GGT, ALB, GLB, DBIL, and IBIL. Retained n indicates the sample size after index-specific outlier exclusion. Model 3 used model-specific complete cases (SIRI, n=1,191; SII, n=1,195; AISI, n=1,194). OR, odds ratio; CI, confidence interval; NAFLD, nonalcoholic fatty liver disease; SIRI, systemic inflammation response index; SII, systemic immune-inflammation index; AISI, aggregate index of systemic inflammation.

**Table S16.** Sensitivity analysis of the association between Lp(a) ≥300 mg/L and the risk of NAFLD (Non-NAFLD vs NAFLD).

| Model | NAFLD status | | | | |
| --- | --- | --- | --- | --- | --- |
|  | n | Non-NAFLD, n (%) | NAFLD, n (%) | OR (95% CI) | *P-value* |
| Model 1 | 2349 | 763 (32.5) | 1586 (67.5) | 0.43 (0.36–0.52) | <0.001 |
| Model 2 | 2349 | 763 (32.5) | 1586 (67.5) | 0.48 (0.39–0.59) | <0.001 |
| Model 3 | 2349 | 763 (32.5) | 1586 (67.5) | 0.50 (0.41–0.62) | <0.001 |

**Note:** Elevated Lp(a) was defined as Lp(a) ≥300 mg/L. NAFLD was coded as the event and Non-NAFLD as the reference outcome. Missing covariate data were handled using multiple imputation by chained equations, generating 30 imputed datasets. Logistic regression was performed separately in each dataset, and regression coefficients and standard errors were pooled using Rubin’s rules. Model 1 was unadjusted. Model 2 was adjusted for age, sex, hypertension, and BMI. Model 3 was further adjusted for diabetes, ALT, AST, TC, TG, HDL-C, LDL-C, UA, eGFR, GGT, ALB, GLB, DBIL, and IBIL. Analyses were restricted to 2,349 participants with valid Lp(a) measurements because Lp(a) was not imputed. The OR compares Lp(a) ≥300 mg/L with Lp(a) <300 mg/L for the odds of NAFLD. OR, odds ratio; CI, confidence interval; NAFLD, nonalcoholic fatty liver disease; Lp(a), lipoprotein(a).
